# Amniotic Membrane Preparation Crucially Affects Its Broad-Spectrum Activity Against Uropathogenic Bacteria

**DOI:** 10.3389/fmicb.2020.00469

**Published:** 2020-03-24

**Authors:** Taja Železnik Ramuta, Marjanca Starčič Erjavec, Mateja Erdani Kreft

**Affiliations:** ^1^Institute of Cell Biology, Faculty of Medicine, University of Ljubljana, Ljubljana, Slovenia; ^2^Department of Biology, Biotechnical Faculty, University of Ljubljana, Ljubljana, Slovenia

**Keywords:** amniotic membrane, uropathogenic bacteria, antimicrobial effect, broad-spectrum, microscopy, ultrastructure, homogenate, patches

## Abstract

Urinary tract infections are among the most common bacterial infections in humans. Moreover, they are highly recurrent and increasingly often resistant to antibiotics. The antimicrobial properties of the amniotic membrane (AM), the innermost layer of fetal membranes, have been briefly reported in the literature, however, the results of published studies are often inconsistent and unclear; moreover, its effect on uropathogenic bacteria has not yet been investigated. Further, there is no data in the literature about the effect of AM preparation and storage on its antimicrobial properties. To examine the impact of several preparation procedures on the antimicrobial properties of AM, we prepared patches and homogenates of fresh (fAM) and cryopreserved (cAM) human AM and tested them on 14 selected Gram-positive and Gram-negative uropathogenic bacteria. By employing novel antimicrobial efficiency assays we showed that fAM and cAM homogenates have broad-spectrum antimicrobial activity against all here tested uropathogenic bacteria, except for *Serratia marcescens*. Moreover, they had a potent effect also on the multiple-resistant clinical strains of uropathogenic *Escherichia coli*. Interestingly, the patches of fAM and cAM had no antimicrobial effect on any of the tested strains. We therefore prepared and stored AM patches according to the standard procedure for clinical use in ophthalmology, which includes the cryopreservation of antibiotic-treated AM, and performed antimicrobial efficiency assays. Our findings suggest that the ultrastructure of AM patches could enable the retention of added antibiotics. In addition, we also prepared gentamicin-resistant uropathogenic *E. coli* strains, which confirmed that the antimicrobial effect of antibiotic-treated AM patches can be attributed to the antibiotic alone. To summarize, here we describe novel protocols for preparation and storage of AM to ensure the preservation of its antimicrobial factors. Moreover, we describe the mechanism of AM retention of antibiotics, based on which the AM could potentially be used as a drug delivery vehicle in future clinically applicable approaches.

## Introduction

Amniotic membrane, the innermost layer of fetal membranes, surrounds the developing fetus and forms the amniotic cavity. The 0.02–0.5 mm thick membrane is composed of a monolayer of amniotic epithelial cells, basal lamina and the avascular stroma, which consists of the compact layer, layer of amniotic mesenchymal stromal cells and spongy layer. AM is abundant in different collagens, proteoglycans and glycoproteins (Niknejad et al., 2008; Rocha and Baptista, 2015; Lee et al., 2016). During pregnancy, the AM contributes to prevention of intrauterine infection, which is critical for proper fetal development (King et al., 2007; PrabhuDas et al., 2015).

The use of AM in clinical practice is increasing. AM has already been recognized as an antimicrobial agent in dermatology for treating chronic wounds (Mohammadi et al., 2013; ElHeneidy et al., 2016; Sant’Anna et al., 2016). Currently, there are two completed clinical trials and 1 clinical trial in running, registered on the NIH Clinical Trials website, which are studying the antimicrobial potential of AM or AM-derived cells in dental medicine (chronic periodontitis) and dermatology (burns, complex wounds). However, none of the clinical studies are testing the antimicrobial effect of the AM in the field of urology (ClinicalTrials.gov).

Urinary tract infections (UTIs) are clinically defined by the presence of a significant number of bacteria in the urine together with accompanying symptoms (Najar et al., 2009). UTIs are among the most common bacterial infections in humans, yearly affecting 150 million people worldwide (Stamm and Norrby, 2001), and they are also highly recurrent (Foxman, 2010, 2014; O’Brien et al., 2015). UTIs occur eight times more often in women than in men, and approximately 50–60% of women report at least one UTI in their lifetime (Foxman et al., 2000; Rahn, 2008; Al-Badr and Al-Shaikh, 2013). Uncomplicated UTIs typically affect otherwise healthy individuals, who do not have structural or neurological urinary tract abnormalities. Complicated UTIs are defined as infections, which develop in patients with structurally or functionally abnormal urinary tract system (Hooton, 2012; Chen et al., 2013; Levison and Kaye, 2013; Flores-Mireles et al., 2015; Majeed et al., 2017). UTIs are caused by Gram-positive and Gram-negative bacteria and fungi. Both complicated and uncomplicated UTIs are most commonly caused by uropathogenic *Escherichia coli* (UPEC) and also often by *Klebsiella pneumoniae*, *Enterococcus* spp., *Staphylococcus aureus*, *Proteus mirabilis*, *Pseudomonas aeruginosa*, group B *Streptococcus*, and *Candida* spp. (Nielubowicz and Mobley, 2010; Levison and Kaye, 2013; Foxman, 2014; Flores-Mireles et al., 2015). Some of UTI-causing agents are becoming progressively more problematic due to an increase of antibiotic resistance and therefore there is an urgent need for the development of new antimicrobial agents (Majeed et al., 2017; Zaman et al., 2017).

Several studies (Talmi et al., 1991; Kjaergaard et al., 2001; Wang et al., 2012; Zare Bidaki et al., 2012; Tehrani et al., 2013; Parthasarathy et al., 2014; Mao et al., 2016, 2017; Yadav et al., 2017; Mao et al., 2018) reported antimicrobial effect of AM on different bacteria (*E. coli*, *S. aureus*, *P. aeruginosa*, *Streptococcus pneumoniae*, *Staphylococcus saprophyticus* etc.) and fungi (*Blastomyces albicans*, *Fusarium solani*, *Aspergillus fumigatus*, *A. niger*, *A. nidulans*). Since the investigation of antimicrobial properties of AM is a developing field, the protocols for preparation and storage of AM are not clearly described and standardized; moreover, the opposite effects of AM on the same bacterial strains have been reported.

Recently we showed that the manner of AM preparation affects its antimicrobial activity (Šket et al., 2019). However, to the best of our knowledge, the impact of different protocols for preparation of AM on the antimicrobial effect on uropathogenic bacteria *in vitro* has not yet been investigated in detail. The aim of this study was therefore to investigate the effect of various procedures of preparation and storage of AM on its antimicrobial effect against several clinical uropathogenic *E. coli* strains: DL88, DL90, DL94, DL101, DL102, isolated from patients with UTI (Rijavec et al., 2006; Starčič Erjavec et al., 2007) and also on several other potentially uropathogenic bacteria, including *S. aureus*, *S. saprophyticus*, *P. mirabilis*, *K. pneumoniae*, *Morganella morganii*, *Providencia rettgeri*, *Enterobacter* spp., and *Serratia marcescens*.

## Materials and Methods

### Microorganisms

Bacterial strains used in this study are listed in Table 1. Additional information about the serotype, phylogenetic group and subgroup, patient’s gender, virulence-associated genes, and antibiotic resistance of UPEC clinical strains are listed in Supplementary Table 1. Overnight cultures were cultured in liquid Luria-Bertani (LB) broth (Formedium, United Kingdom). Gentamicin-resistant strains were grown on LB agar plates with added gentamicin (25 μg/ml). All antimicrobial efficiency assays were carried out using liquid Muller-Hinton broth and Muller-Hinton agar plates (Formedium, United Kingdom). When cultured in liquid LB broth, the cultures were grown with aeration (180 rpm).

**TABLE 1:** List of bacterial strains used in the experiments.

Strains	Relevant genotype and/or phenotype features	Gram stain	References/source
*Escherichia coli* Top10 pMW2-Gm^r^	F– *mcr*A Δ(*mrr*-*hsd*RMS-*mcr*BC) Φ80*lac*ZΔM15 Δ*lac*X74 *rec*A1 *ara*D139 Δ(*ara leu*)7697 *gal*U *gal*K *rps*L *end*A1 *nup*G pMW2-Gm^r^	Gram-negative	Kužnik, 2017
*Escherichia coli* DH5α	Φ80d*lacZ*ΔM15 Δ(*lacZYA-argF*)U169 *endA1 recA1 hsdR17 deoR thi-1 supE44 gyrA96 relA1*	Gram-negative	Invitrogen, United States
*Escherichia coli* DL88	w.t. strain	Gram-negative	Mulec et al., 2002; Rijavec et al., 2006
*Escherichia coli* DL90	w.t. strain	Gram-negative	Rijavec et al., 2006; Starčič Erjavec et al., 2007
*Escherichia coli* DL94	w.t. strain	Gram-negative	Mulec et al., 2002; Rijavec et al., 2006
*Escherichia coli* DL101	w.t. strain	Gram-negative	Mulec et al., 2002; Rijavec et al., 2006
*Escherichia coli* DL102	w.t. strain	Gram-negative	Mulec et al., 2002; Rijavec et al., 2006
*Escherichia coli* DL88-Gm^r^	DL88 Gm^r^	Gram-negative	This study
*Escherichia coli* DL90-Gm^r^	DL90 Gm^r^	Gram-negative	This study
*Klebsiella pneumoniae* JM75	w.t. strain	Gram-negative	Mulec et al., 2002
*Proteus mirabilis* JM80	w.t. strain	Gram-negative	Mulec et al., 2002
*Serratia marcescens* EXB V 15	w.t. strain	Gram-negative	“Ex Culture Collection,” SI
*Providencia rettgeri* EXB L-365	w.t. strain	Gram-negative	“Ex Culture Collection,” SI
*Morganella morganii* EXB L-367	w.t. strain	Gram-negative	“Ex Culture Collection,” SI
*Staphylococcus saprophyticus* EXB V 56	w.t. strain	Gram-positive	“Ex Culture Collection,” SI
*Staphylococcus aureus* EXB V 110	w.t. strain	Gram-positive	“Ex Culture Collection,” SI
*Enterobacter* sp. EXB V 11	w.t. strain	Gram-negative	“Ex Culture Collection,” SI

**Escherichia coli* Top10 pMW2-Gm^*r*^ was used for the preparation of gentamicin-resistant strains UPEC DL88-Gm^*r*^ and UPEC DL90-Gm^*r*^. All other strains were used in antimicrobial efficiency assays to test the antimicrobial properties of AM.*

### Preparation of Gentamicin-Resistant UPEC Strains

We prepared gentamicin-resistant UPEC DL88 and DL90 strains by transformation of the pMW2-Gm^r^ plasmid, a plasmid harboring a gentamicin resistance gene (Kužnik, 2017). Isolation of plasmid pMW2-Gm^r^ from *E. coli* Top10 strain was performed by using standard methods (Sambrook et al., 1989). Briefly, overnight bacterial culture *E. coli* Top10 harboring the pMW2-Gm^r^ plasmid in liquid LB broth was centrifuged for 1 min at 18,000 *g*. The pellet was resuspended in solution I (50 mM glucose/58 mM Tris HCl pH 8.0, 10 mM EDTA pH 8.0) and then solution II was added [200 mM NaOH, 1% SDS (w/v)]. After 5 min incubation on ice, solution III was added (5M potassium acetate pH 5.5), followed by another 5-min incubation on ice. After 10 min centrifugation at 16,000 *g*, the supernatant was transferred into a new tube and the same volume of extraction buffer (phenol, chloroform, isoamyl alcohol, 25:24:1) was added. The solution was mixed and centrifuged at 18,000 *g* and the upper phase was transferred into a new tube. After adding 96% ethanol, the solution was centrifuged at 13,500 *g* and the pellet was briefly rinsed with 70% ethanol, air-dried and resuspended in TE buffer (10 mM Tris HCl pH 8.0, 1 mM EDTA) with RNase A (100 μg/ml).

Next, we prepared the UPEC DL88 and DL90 strains for electroporation. Briefly, 1 ml of the overnight culture of each strain was first inoculated in 100 ml of LB broth and grown at 37°C with aeration (180 rpm) until they reached the OD (600) 0.5–0.6. Then the cultures were incubated on ice for 15 min and centrifuged for 20 min at 5000 *g* at 4°C. Then the supernatants were discarded and the pellets were resuspended in 45 ml of sterile distilled H_2_O, vortexed and centrifuged again for 20 min at 5000 *g* at 4°C. After centrifugation the pellets were resuspended in 10% chilled glycerol, centrifuged again for 20 min at 5000 *g* at 4°C and then resuspended in 10% glycerol and stored at −80°C until use.

UPEC DL88 and DL90 strains were electroporated using the Electroporator 2510 (Eppendorf, Germany) according to the manufacturer’s protocols. Briefly, to prepare gentamicine-resistant UPEC strains, 2 μl of DNA of the pMW-Gm^*r*^ plasmid were added to 40 μl of electrocompetent UPEC DL88 and UPEC DL90 strains and incubated on ice for 2 min. Next, the samples were electroporated at 1700 V for 5 ms and then incubated in SOC medium for 30–60 min with moderate shaking at 37°C. To ensure the appropriate selection, the transformed bacteria were then grown on LB agar plates supplemented with gentamicin (25 μg/ml).

### Ethics Statement

The study on human AM was approved by the National Medical Ethics Committee of the Republic of Slovenia and prepared following the standard procedure (Mikek et al., 2004; Jerman et al., 2014). Briefly, 9 placentas were obtained with written informed consent at the time of elective cesarean sections from healthy volunteers. All volunteers were serologically negative for HIV, syphilis, and hepatitis B and C.

### Preparation of fAM and cAM

The placentas were rinsed with sterile phosphate-buffered saline (PBS). The AM was manually separated from chorion, cut into pieces of approximately 3 cm × 3 cm and stored in PBS at 4°C for a maximum of 6 h before use fAM or cryopreserved in PBS at −80°C for 1 week before use cAM. All cryopreserved samples went only through one freeze-thaw cycle.

#### Preparation of Cryopreserved AM Patches, Which Came Into Contact With Antibiotics (cAM + atb)

The placentas were briefly rinsed with physiological saline, containing 50 μg/ml penicillin, 50 μg/ml streptomycin, 100 μg/ml neomycin, and 2.5 μg/ml amphotericin B. The AM was manually separated from chorion, cut into pieces of approximately 3 cm × 3 cm, and cryopreserved in the Modified Eagle’s medium (Gibco) and glycerol in a volume ration 1:1 with added gentamicin (25 μg/ml) at −80°C for 1 week before use.

### Preparation of fAM and cAM Homogenates

Phosphate-buffered saline was added to patches of fAM or cAM (volume ratio 3:1) and homogenized in a homogenizer (Russell Hobbs, 21350-56, 300 W) for 3–4 min (Šket et al., 2019). Homogenate was stored at 4°C for a maximum of 6 h before use (fAM homogenate) or cryopreserved at −80 or at −20°C (cAM homogenate). cAM homogenates were used after 1 or 10 weeks of cryopreservation. All cryopreserved samples used in the experiments went only through one freeze-thaw cycle.

#### Preparation of cAM + atb (1 Week at −80°C) Homogenate

Patches of cAM + atb were thawed and rinsed with PBS for 15 min (during which PBS was changed 7–10 times) to remove cryopreservation medium. PBS was added to patches of cAM + atb (volume ratio 3:1) and homogenized in a homogenizer (Russell Hobbs, 21350-56, 300 W) for 3–4 min. Homogenate was cryopreserved at −80°C and used after 1 week of cryopreservation [cAM homogenate (1 week at −80°C)]. All cryopreserved samples used in the experiments went only through one freeze-thaw cycle.

### Antimicrobial Efficiency Assay

Bacteria were inoculated in LB broth and grown with aeration (180 rpm) overnight at 37°C.

#### Antimicrobial Efficiency Assay With AM Patches Embedded in Soft Agar

fAM and cAM were briefly rinsed in PBS and cut into 1 cm × 1 cm patches and placed on the Muller-Hinton agar plate. cAM + atb were rinsed with PBS for 15 min (during which PBS was changed 7–10 times) to remove cryopreservation medium, cut into 1 cm × 1 cm patches and placed on the Muller-Hinton agar plate. Muller-Hinton soft agar was first cooked, cooled to 48°C and then inoculated with 100 μl of overnight culture and poured over the Muller-Hinton agar plate with fAM/cAM (1 week at −80°C)/cAM + atb patch. Plates were incubated at 37°C for 24 h (Supplementary Figure 1).

#### Antimicrobial Efficiency Assay With AM Patches on Solid Agar

Pieces of cAM + atb were rinsed with PBS for 15 min (during which PBS was changed 7–10 times) to remove cryopreservation medium and cut into 1 cm × 1 cm patches. Overnight culture (100 μl) was plated on Muller-Hinton agar using sterile beads. After 15 min of incubation at room temperature, a cAM + atb patch was placed on the plate. Plates were incubated at 37°C for 24 h (Supplementary Figure 1).

#### Antimicrobial Efficiency Assay With AM Homogenate

Muller-Hinton soft agar was first cooked, cooled to 48°C and then inoculated with 100 μl of overnight culture and poured over the Muller-Hinton agar plate. After 15 min of incubation at room temperature, three-times of 5 μl and three-times of 10 μl of fAM/cAM/cAM + atb homogenate were placed on the agar plate. Plates were incubated at 37°C for 24 h (Supplementary Figure 1).

### Statistical Analysis

All data were calculated from 3 to 6 biological samples of AM and 6–30 technical repeats all together for each strain for each assay and are presented as mean ± standard error. Statistical analysis was performed using a two-tailed Student’s *t*-test and one-way ANOVA. *p*-values of < 0.05 were considered statistically significant. All statistical analyses were performed using GraphPad Prism 6 (GraphPad Software, Inc., La Jolla, CA, United States).

### Transmission and Scanning Electron Microscopy

Samples of fAM and cAM patches were analyzed by transmission and scanning electron microscopy. Samples were prepared as described previously (Jerman et al., 2014; Višnjar et al., 2017a, b). Briefly, samples were fixed with 4% (w/v) paraformaldehyde and 2.5% (v/v) glutaraldehyde in a 0.1M cacodylate buffer (pH 7.4) for 2 h and 45 min at 4°C. Specimens for transmission electron microscopy were rinsed overnight in 0.1M cacodylate buffer at 4°C and then post-fixed in 2% (w/v) osmium tetroxide for 1 h at 4°C, dehydrated in a graded series of ethanol and embedded in Epon (Serva Electrophoresis, Germany). Ultrathin sections were contrasted with uranyl acetate and lead citrate and examined with Philips CM100 electron microscope. The specimens for scanning electron microscopy were rinsed overnight in 0.2M cacodylate buffer at 4°C and then post-fixed in 1% osmium tetroxide in 0.2M cacodylate buffer for 90 min at 4°C. Then they were dehydrated through a graded series of ethanol and acetone. Afterward, specimens were immersed in HMDS (hexamethyldisilazane, Sigma), air-dried, and sputter-coated with gold, and examined at 30 kV with Tescan Vega 3 scanning electron microscope.

### Data Availability

The authors declare that all data supporting the findings of this study are available within the manuscript and its Supplementary Material. All information about the materials and methods used are available also in the Protocols.io database (Ramuta et al., 2019).

## Results

### Patches of fAM and cAM Have No Antimicrobial Effect on Tested Strains Unless Prepared and Stored According to the Standard Procedure for Clinical Use of AM

**FIGURE 1:**
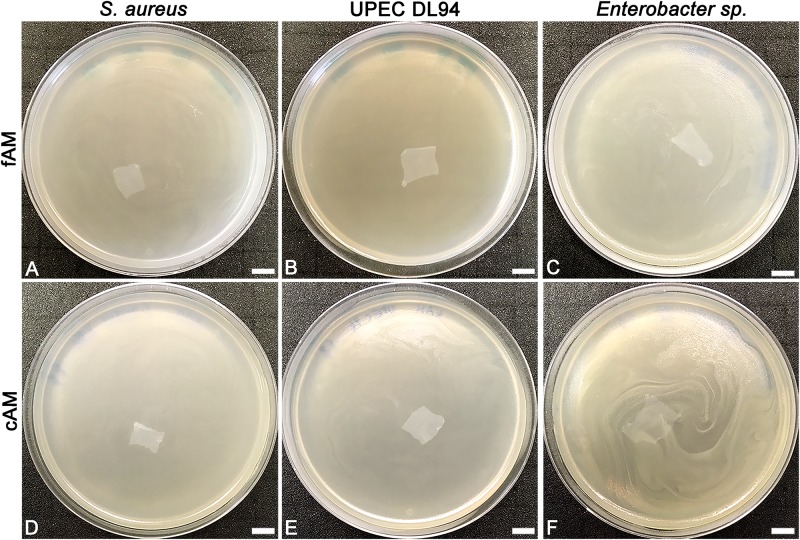
The effect of fresh (fAM) and cAM patches on *Staphylococcus aureus*, UPEC DL94 and *Enterobacter* sp. (**A–C**) fAM patches and (**D–F**) cAM patches have no antimicrobial effect on tested strains. These results were obtained by employing antimicrobial efficiency assay with AM patches embedded in soft agar. Three independent replications of experiments using three biological samples were conducted and three technical repeats were performed for each condition. Scale bars: 10 mm.

**FIGURE 2:**
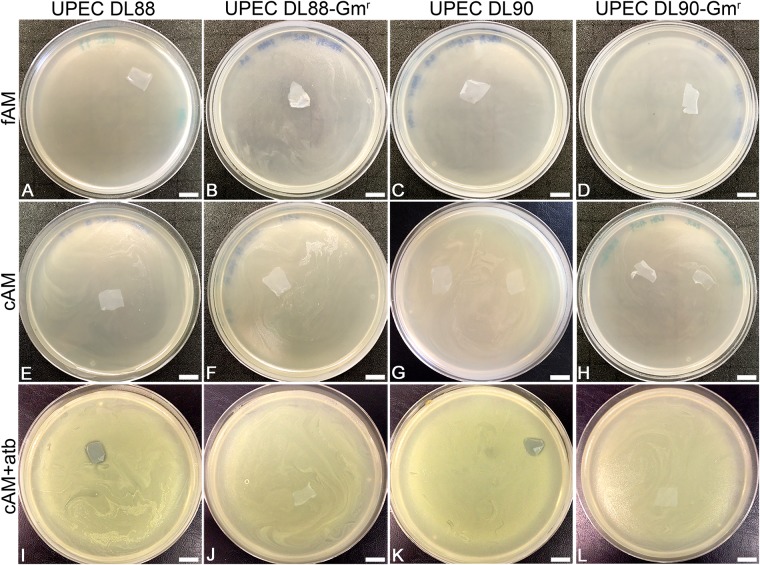
The antimicrobial effect of fresh (fAM), cryopreserved (cAM) and cAM + atb patches on UPEC DL88 and UPEC90 and gentamicin-resistant UPEC strains (UPEC DL88-Gm^r^, UPEC DL90-Gm^r^). fAM **(A–D)** and cAM **(E–H)** patches have no antimicrobial effect on UPEC strains and gentamicin-resistant UPEC strains. cAM + atb patches have antimicrobial effect on **(I)** UPEC DL88 and **(K)** UPEC DL90 strains, however, they have no antimicrobial effect on **(J)** UPEC DL88-Gm^r^ and **(L)** UPEC DL90-Gm^r^ strains. These results were obtained by employing antimicrobial efficiency assay with AM patches embedded in soft agar. Three independent replications of experiments using three biological samples were conducted and three technical repeats were performed for each condition. Scale bars: 10 mm.

In clinical practice, the AM is most commonly used in the form of patches. Here we prepared patches (1 cm × 1 cm) of fresh AM and used them immediately (fAM patches) or stored at −80°C for 1 week before usage (cAM patches). Patches were embedded in soft agar, which was previously inoculated with tested strains. After 24 h of incubation at 37°C, agar plates were examined. All plates in all assays (three biological samples of AM and nine technical repeats for each tested strain) were overgrown with bacteria (i.e., confluent growth), showing that fAM and cAM patches have no inhibitory effect on the growth of any here tested strain (Figures 1, 2A–H,J,L).

**FIGURE 3:**
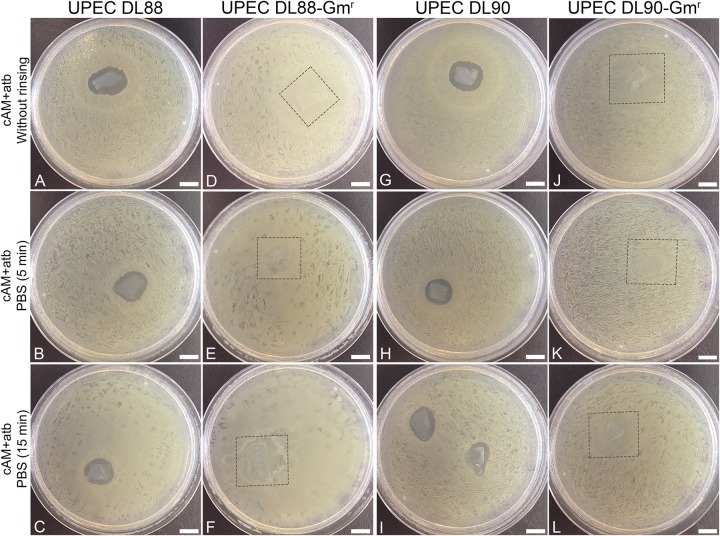
Rinsing cAM + atb patches in phosphate-buffered saline (PBS) decreases the antimicrobial effect of cAM + atb patches on UPEC DL88 and UPEC DL90 strains. **(A–C,G–I)** Increased rinsing time of cAM + atb patches results in a decrease of antimicrobial zones in case of UPEC DL88 and UPEC DL90. **(D–F,J–L)** cAM + atb patches have no antimicrobial effect on UPEC DL88-Gm^r^ and UPEC DL90-Gm^r^. These results were obtained by employing antimicrobial efficiency assay with AM patches on solid agar. Two independent replications of experiments using two biological samples were conducted and three technical repeats were performed for each condition. Square frame – the location of cAM + atb patch, overgrown with tested strain. Scale bars: 10 mm.

**FIGURE 4:**
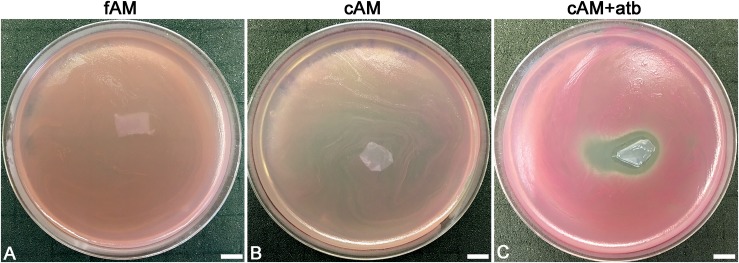
Patches of amniotic membrane could serve as a drug-delivery tool. **(A,B)** Patches of fresh (fAM) and cryopreserved AM (cAM) have no antimicrobial effect on *S. marcescens*. **(C)** When patches of AM are impregnated with an antibiotic (cAM + atb), strong antimicrobial effect on *S. marcescens* is seen. Three independent replications of experiments using three biological samples were conducted and three technical repeats were performed for each condition. These results were obtained by employing antimicrobial efficiency assay with AM patches embedded in soft agar. Scale bars: 10 mm.

To investigate whether AM, prepared and stored according to the procedure for clinical use of AM, has antimicrobial activity, antimicrobial efficiency assays were performed with cAM patches prepared according to the procedure for clinical use in ophthalmology (Jirsova and Jones, 2017). During such preparation AM is briefly washed with antibiotics and antimycotics (50 μg/ml penicillin, 50 μg/ml streptomycin, 100 μg/ml neomycin, 2.5 μg/ml amphotericin B) and then stored in culture medium with gentamicin (25 μg/ml), which causes impregnation of cAM patches with gentamicin (cAM + atb). In all performed antimicrobial efficiency assays with cAM + atb patches, the cAM + atb patches exhibited the antimicrobial effect on all here tested strains, including *S. marcescens* (Figures 2I,K, 3A–C,G–I, 4C).

### The Antimicrobial Effect of cAM + atb Patches Is Entirely Due to Impregnation With an Antibiotic

As we hypothesized that the observed antimicrobial efficiency in the cAM + atb assays was only due to the antibiotic gentamicin in the cAM + atb patches, gentamicin-resistant UPEC strains DL88 and DL90 were prepared (UPEC DL88-Gm^r^ and UPEC DL90-Gm^r^) and tested. As expected, cAM + atb patches showed no antimicrobial effect on UPEC DL88-Gm^r^ and UPEC DL90-Gm^r^ strains (Figures 2, 3).

To further confirm the role of the antibiotic gentamicin for the observed antimicrobial effect, rinsing of cAM + atb patches before use was performed. The obtained results showed that the increase in rinsing time resulted in decreased antimicrobial zones. As even 5 min rinsing time [during which phosphate-buffered saline (PBS) was changed 3–5 times] and 15 min rinsing time (during which PBS was changed 7–10 times) did not result in a profound decrease of the antimicrobial zone, it can be assumed that the antibiotics are well-retained by AM patches (Figure 3).

Furthermore, the ability of AM to contain antibiotics was additionally confirmed by testing the antimicrobial effect of cAM + atb patches on *S. marcescens*. While fAM and cAM patches had no antimicrobial effect on *S. marcescens* (Figures 4A,B), cAM + atb patches had a potent antimicrobial effect (Figure 4C).

### High Retention of Antibiotics in AM Patch Could Be Attributed to Its Ultrastructure

**FIGURE 5:**
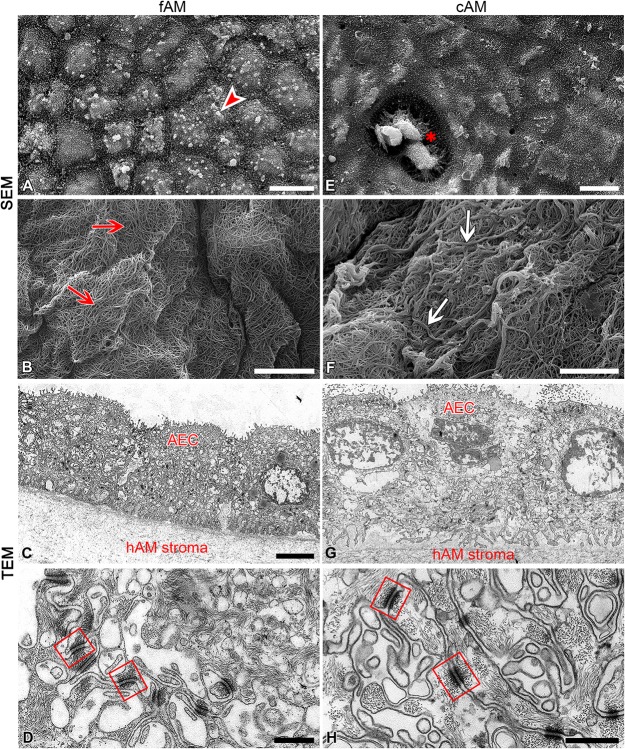
Cryopreservation affects the ultrastructure of amniotic membrane. **(A–D)** Ultrastructure of fAM. Amniotic epithelial cells (AEC) are well-connected with surrounding cells and basal lamina **(A,C,D)**, and fibers of fAM stroma **(B)** were tightly interwoven (red arrows). **(E–H)** Ultrastructure of cAM. The apical surface is partly damaged, the remaining AEC are well-connected with surrounding cells and basal lamina **(E,G,H)**, and fibers of cAM stroma are forming thicker strands of fibers (**F**; white arrows). Bulbous forms on apical microvilli of amniotic epithelial cells of fAM (arrowhead), area of exposed basal lamina due to removal of AEC (**E**; asterisk), well-developed desmosomes (squares) connecting AEC. Three independent replications of experiments using three biological samples of fAM were conducted and five independent replications of experiments using five biological samples of cAM were performed. Scale bars: **(A,E)** 10 μm; **(B,C,F,G)** 5 μm; **(D,H)** 500 nm.

To determine whether high antimicrobial activity of antibiotic-impregnated AM patches could be explained by the ultrastructure of AM, analyses with scanning and transmission electron microscopes were performed. AM is composed of a monolayer of cuboidal amniotic epithelial cells, the basal lamina and avascular stroma. Amniotic epithelial cells in fAM patches were well-connected with surrounding cells with desmosomes and basal lamina with hemidesmosomes. Their apical microvilli were fairly uniform and dense, but they also showed some variation in size and shape and occasionally generated branched or bulbous forms (Figures 5A–D). The fibers of fAM stroma were tightly interwoven (Figure 5B). However, cryopreservation affected the ultrastructure of cAM patch on several levels. First, the apical surface of cAM patch was damaged, in some places basal lamina was exposed due to the removal of amniotic epithelial cells and we also detected few regions, where amniotic epithelial cells were desquamated (Figure 5E). The remaining cells were connected with surrounding cells and basal lamina through desmosomes and hemidesmosomes. After cryopreservation, the microvilli still showed some variation in size and shape, they occasionally generated branched forms, but on some cells, they appeared elongated and agglutinated (Figures 5E–H). The individual fibers of cAM stroma agglutinated and formed thicker strands of fibers (Figure 5F). We hypothesize that the high antimicrobial activity of cAM + atb might be due to the high retention of gentamicin in this preparation conferred by the unique ultrastructure of AM. Our results and results of Mencucci et al. (2006) show that cAM + atb patches allow high retention of antibiotics. Moreover, we show that the ultrastructure of fAM + atb patches was even better preserved, indicating that further studies on fAM + atb patches’ potential for clinical use are required.

### Homogenates of Fresh and Cryopreserved Amniotic Membrane Have a Powerful Antimicrobial Effect on Tested Strains

**FIGURE 6:**
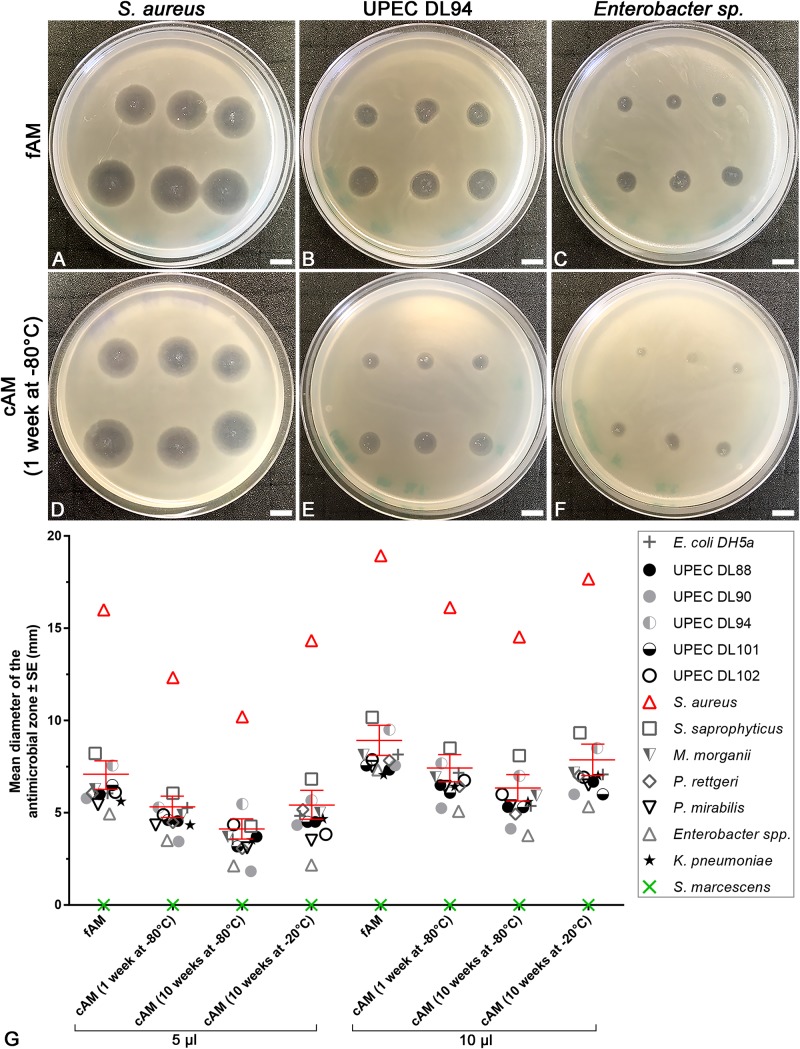
The range of antimicrobial effect of fresh (fAM) and cAM homogenates varies between tested strains. **(A–F)** Homogenates of fAM and cAM have antimicrobial effect on *S. aureus*, UPEC DL94 and *Enterobacter* sp. The range of antimicrobial effect of fAM and cAM (1 week at –80°C) homogenates varies, namely between strong (*S. aureus*)/moderate (UPEC DL94)/minor (*Enterobacter* sp.) antimicrobial effect. Furthermore, fAM homogenate inhibits the growth of tested strains **(A–C)**, while cAM (1 week at –80°C) homogenate inhibits the growth of *S. aureus* and decreases the growth of UPEC DL94 and *Enterobacter* sp. **(D–F)**. The quantity of fAM and cAM (1 week at –80°C) homogenates used was 5 μl (upper rows) and 10 μl (lower rows). Scale bars: 10 mm. **(G)** The average antimicrobial effect of fAM and cAM homogenates on all tested strains. Larger volumes of homogenates (10 μl) have greater antimicrobial effect than smaller volumes (5 μl). For fAM each point represents the mean diameter of the antimicrobial zone ± SE (mm) for three independent replications of experiments using three biological samples; each experiment was performed in six technical repeats for each strain. For cAM (1 week at –80°C) each point represents the mean diameter of the antimicrobial zone ± SE (mm) for four independent replications of experiments using four biological samples; each experiment was performed in three to six technical repeats for each strain. For cAM (10 weeks at –80°C) each point represents the mean diameter of the antimicrobial zone ± SE (mm) for five independent replications of experiments using five biological samples; each experiment was performed in six technical repeats for each strain. For cAM (10 weeks at –20°C) each point represents the mean diameter of the antimicrobial zone ± SE (mm) for one to three independent replications of experiments using one to three biological samples; each experiment was performed in three to six technical repeats for each strain. Bars in red represent the mean diameter of the antimicrobial zone ± standard error (mm) of all tested strains.

To examine whether the intrinsic antimicrobial factors can be released from AM by homogenization, homogenates of fresh (fAM) and cAM were prepared and antimicrobial efficiency assays were performed. Namely, fAM and cAM homogenates were pipetted on soft agar, previously inoculated with tested strains. After 24 h of incubation at 37°C, the antimicrobial effect of AM homogenates on tested strains was examined. The assays were performed with 5 or 10 μl of AM homogenate. Further, the effects of various conditions for storage of AM homogenates were evaluated, as the antimicrobial effect of AM homogenates was tested after 1 or 10 weeks of storage at −80°C [cAM (1 week at −80°C), cAM (10 weeks at −80°C) homogenates] and after 10 weeks of storage at −20°C [cAM (10 weeks at −20°C) homogenate]. fAM and cAM homogenates exhibited antimicrobial effect on all here tested strains, with the exception of *S. marcescens*. Namely, the susceptible strains are: *E. coli* DH5α, clinical UPEC strains DL88, DL90, DL94, DL101, DL102, *K. pneumoniae*, *P. mirabilis*, *P. rettgeri*, *M. morganii*, *S. saprophyticus*, *S. aureus* and *Enterobacter* sp. Moreover, homogenate of fAM inhibited the growth of all susceptible strains, whereas cAM homogenates inhibited or only decreased the growth of susceptible strains (Supplementary Table 2 and Figure 6).

The extent of the antimicrobial effect varied between tested strains. Namely, the application of AM homogenates resulted in strong (e.g., *S. aureus*), moderate (e.g., UPEC DL94) or minor antimicrobial effect (e.g., *Enterobacter* sp.). The analysis of variance (ANOVA) was performed to analyze the extent of antimicrobial effect of various AM homogenates within the same bacterial strain and between all tested strains. Our results show that even when only 5 μl of AM homogenates were applied, a prominent antimicrobial effect was detected. Namely, the mean diameter of the antimicrobial zone for all susceptible strains was for fAM homogenate 7.0 ± 0.2 mm, cAM (1 week at −80°C) homogenate 5.2 ± 0.2 mm, cAM (10 weeks at −80°C) homogenate 4.0 ± 0.3 mm, and for cAM (10 weeks at −20°C) homogenate 5.3 ± 0.4 (Supplementary Tables 2–5).

Further analyses showed that storage conditions crucially affected the antimicrobial activity of AM homogenates. First, we applied 5 μl of AM homogenates. The antimicrobial effect of fAM homogenate was on average for 25% higher than of cAM (1 week at −80°C) and cAM (10 weeks at −20°C) homogenates (Supplementary Table 5). The mean diameter of the antimicrobial zones for all susceptible strains together was in fAM homogenate 7.0 ± 0.2 mm (Supplementary Table 5). Similarly, when applying 10 μl of AM homogenates, the fAM homogenate caused the largest antimicrobial zone in susceptible strains, followed by cAM (1 week at −80°C) and cAM (10 weeks at −20°C) homogenates, while the cAM (10 weeks at −80°C) homogenate caused the smallest antimicrobial effect (Supplementary Table 5). These data underline the importance of choosing the correct storage conditions to preserve the antimicrobial activity of AM homogenates.

The analysis of variance (ANOVA) showed that for all strains, except UPEC DL102 and *Enterobacter* sp., the ranges of antimicrobial zones of cAM (1 week at −80°C) and cAM (10 weeks at −20°C) homogenates were not statistically significantly different between both storage durations (*p* > 0.05). However, when comparing the range of antimicrobial effect of fAM homogenate with the cAM homogenates stored at −80°C for each tested strain separately, ANOVA showed that there was a statistically significant difference in 85% (5 μl) and 62% (10 μl) of all cases (Supplementary Tables 2–4). On the other hand, when comparing the ranges of antimicrobial effect of fAM homogenate with the cAM (10 weeks at −20°C) homogenate for each tested strain separately; a statistically significant difference was lower, found in 50% (5 μl) and 36% (10 μl) of all cases (Supplementary Tables 2–4). If we do not use fAM, then cryopreservation of AM homogenate at −20°C is the most suitable preservation procedure among those we tested, since it preserves most of the intrinsic antimicrobial factors.

### fAM and cAM (1 Week at −80°C) Homogenates Have a Greater Antimicrobial Effect on UPEC Strains Than cAM + atb (1 Week at −80°C) Homogenate

**FIGURE 7:**
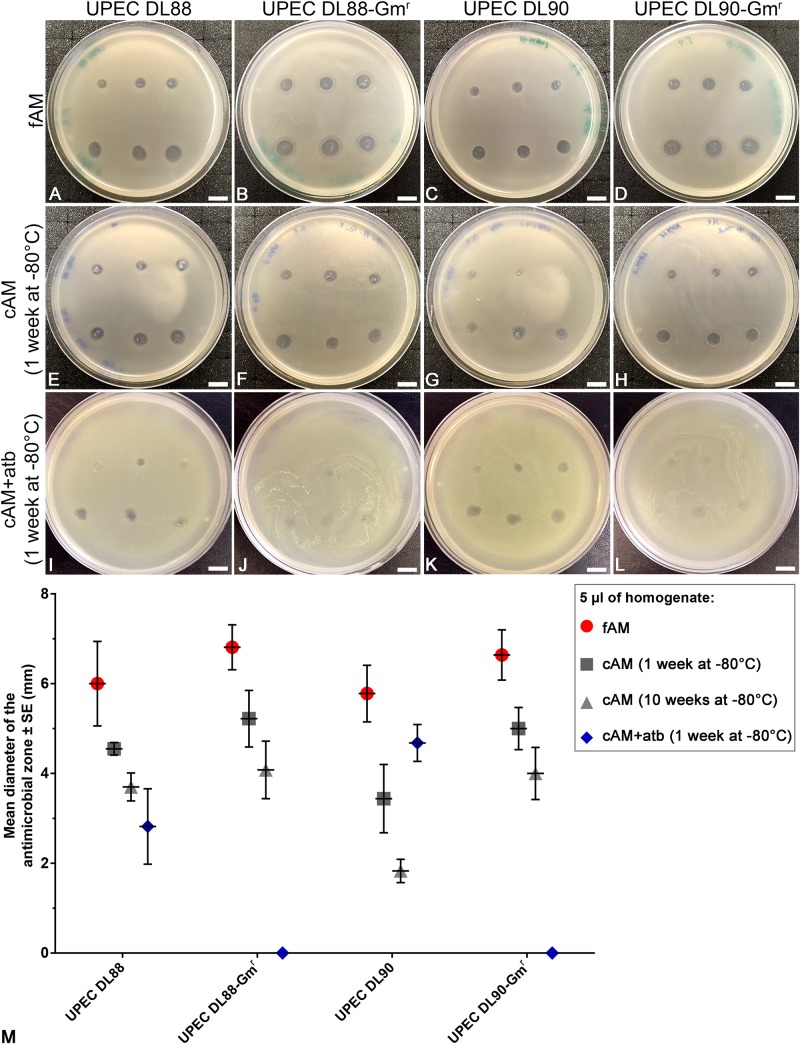
Homogenates of fresh (fAM) and cAM (1 week at –80°C) have greater antimicrobial effect on UPEC strains and gentamicin-resistant UPEC strains than antibiotic-impregnated cAM + atb (1 week at –80°C) homogenate. **(A–H)** fAM and cAM (1 week at –80°C) homogenates have antimicrobial effect on UPEC DL88 and UPEC90 strains and gentamicin-resistant UPEC strains (UPEC DL88-Gm^r^, UPEC DL90-Gm^r^). **(I,K)** cAM + atb (1 week at –80°C) homogenate has antimicrobial effect on UPEC DL88 and UPEC DL90 strains, but not on UPEC DL88-Gmr and UPEC DL90-Gmr strains **(J–L)**. Slight decrease in UPEC DL88-Gm^r^ and UPEC DL90-Gm^r^ growth might be attributed to residual intrinsic antimicrobial factors of AM that were not washed out of AM during preparation and storage. The quantity of fAM, cAM (1 week at –80°C) and cAM + atb (1 week at –80°C) homogenates used was 5 μl (upper rows) and 10 μl (lower rows). Scale bars: 10 mm. **(M)** Each point presents the mean diameter of the antimicrobial zone ± standard error (mm) when testing fAM/cAM (1 week at –80°C)/cAM (10 weeks at –80°C)/cAM + atb (1 week at –80°C) homogenates on UPEC and gentamicin-resistant UPEC strains. The quantity of AM homogenates used was 5 μl. For fAM each point represents the mean diameter of the antimicrobial zone ± SE (mm) for three independent replications of experiments using three biological samples; each experiment was performed in nine technical repeats for each strain. For cAM (1 week at –80°C) each point represents the mean diameter of the antimicrobial zone ± SE (mm) for three independent replications of experiments using three biological samples; each experiment was performed in three technical repeats for each strain. For cAM (10 weeks at –80°C) each point represents the mean diameter of the antimicrobial zone ± SE (mm) for three independent replications of experiments using three biological samples; each experiment was performed in six to nine technical repeats for each strain.

Next, to determine whether the antimicrobial effect of antibiotic-impregnated AM on UPEC strains is comparable with the antimicrobial effect of intrinsic antimicrobial factors of AM, we prepared homogenates of fAM, cAM (1 week at −80°C) and cAM + atb (1 week at −80°C) and performed antimicrobial efficiency assays. Our results show that fAM and cAM (1 week at −80°C) homogenates had a potent antimicrobial effect on UPEC DL88 and DL90 and also on gentamicin-resistant UPEC strains (UPEC DL88-Gm^r^, UPEC DL90-Gm^r^) (Figures 7A–H). On the other hand, cAM + atb (1 week at −80°C) homogenate had an antimicrobial effect on UPEC DL88 and UPEC DL90 strains (Figures 7I,K), but it was smaller in comparison to the antimicrobial effect of fAM and cAM (1 week at −80°C) homogenates without antibiotic. The inhibitory effect of all tested homogenates was dose-dependent.

When testing the effect of cAM + atb (1 week at −80°C) homogenate on gentamicin-resistant strains UPEC DL88-Gm^r^ and UPEC DL90-Gm^r^, plates were overgrown with tested strains. However, a slight decrease in bacterial growth was detected where the cAM + atb (1 week at −80°C) homogenate was deposited (Figures 7J,L), which could be attributed to residual intrinsic antimicrobial factors of AM that were not washed out of AM during preparation and storage.

The quantification of antimicrobial zones revealed that fAM homogenate has the largest antimicrobial effect. The effect of cAM + atb (1 week at −80°C) homogenate was slightly varied between UPEC DL88 and UPEC DL90 strains, although differences between strains were not statistically significant, and had no antimicrobial effect on gentamicin-resistant strains (Figure 7). We hypothesize that the variations in the antimicrobial effect of cAM + atb (1 week at −80°C) homogenate could be attributed to differences in thickness and structure of cAM + atb patches from which cAM + atb homogenates are prepared. Namely, thicker stroma could retain larger amounts of antibiotics, resulting in a greater antimicrobial effect.

## Discussion

Urinary tract infections have become a staggering burden to health care systems worldwide since their management has become increasingly difficult due to the emergence of antibiotic-resistant uropathogenic bacteria (Foxman, 2014; Magistro and Stief, 2018). Hence, researchers are searching for new antimicrobial agents (Lüthje and Brauner, 2016; Alidjanov et al., 2017; Babikir et al., 2018) and even vaccines (Moriel and Schembri, 2013; Mobley and Alteri, 2015; Magistro and Stief, 2018) to tackle this issue. Antimicrobial peptides are expressed by different human cells, including amniotic epithelial cells. They excrete antimicrobial factors, such as human α-defensins (Svinarich et al., 1997; King et al., 2007), β-defensins (HBD) 1–3 (King et al., 2007), SLPI (secretory leukocyte protease inhibitor) and elafin (Denison et al., 1999; Zhang et al., 2001), which are a part of the innate immune system (King et al., 2007). Additionally, also histones H2A and H2B that possess antimicrobial properties and endotoxin-neutralizing activity, are present in the cytoplasm and on the extracellular surface of amniotic epithelial cells (Kim et al., 2002). Our study demonstrated for the first time that AM has a powerful broad-spectrum antimicrobial effect on Gram-positive and Gram-negative bacteria. Furthermore, we showed that the procedure of preparation and storage crucially affects the extent of the antimicrobial effect of AM.

### Patches of AM Have No Antimicrobial Effect, but They Could Serve as a Drug-Delivery Tool

A few studies (Talmi et al., 1991; Kjaergaard et al., 2001; Wang et al., 2012; Zare Bidaki et al., 2012; Tehrani et al., 2013; Parthasarathy et al., 2014; Mao et al., 2016, 2017, 2018; Yadav et al., 2017) reported antimicrobial effect of AM patches, extracts or conditioned media derived from AM cells on different bacteria (*E. coli*, *S. aureus*, *P. aeruginosa*, *S. pneumoniae*, *S. saprophyticus* etc.) and on fungi (*B. albicans*, *F. solani*, *A. fumigatus*, *A. niger*, *A. nidulans*). However, some of the results from these studies are contradictory, since they showed the opposite effect (the presence or the lack of antimicrobial response) in the same bacterial species.

In a clinical setting, AM is most commonly used in the form of a patch, therefore we first performed antimicrobial efficiency assays using fresh (fAM) and cryopreserved AM (cAM) patches. Surprisingly, patches of fAM and cAM elicited no antimicrobial effect on any of the here tested strains. Bacterial growth was not inhibited under the fAM and cAM patches and no antimicrobial zone was detected. However, AM patches, prepared according to the standard AM preparation procedure for clinical use in ophthalmology (Jirsova and Jones, 2017) and hence stored in cryopreservation medium with gentamicin, possessed antimicrobial efficiency.

To prepare antibiotic-impregnated AM, we chose gentamicin, since it is broadly used for treatment of various conditions, including septicemia, bacterial endocarditis, peritonitis, meningitis, pelvic inflammatory disease, pneumonia and also UTIs. Aminoglycoside antibiotics are due to their antimicrobial efficacy, widespread availability and low cost commonly prescribed, however, they can induce nephrotoxicity, cochleotoxicity and vestibulotoxicity, especially when applied intravenously (Jiang et al., 2017). Since gentamicin is broadly used in clinic and the topical application of the antibiotic presents a lower risk of side effects than a systemic application, we suggest the use of gentamicin-impregnated AM patches should be further investigated also because AM has additional beneficial properties, such as promotion of wound healing (ElHeneidy et al., 2016; Barski et al., 2018) and decrease of fibrosis (Navas et al., 2018).

Next, as our further experiments revealed that the antimicrobial efficiency of such patches was due to gentamicin, consequently we tried to ascertain whether antimicrobial activity of cAM + atb patches might be supported by the unique ultrastructure of AM. Therefore, analyses with scanning and transmission electron microscopes were performed. AM is composed of amniotic epithelial cells, the basal lamina and thick stroma. The latter is rich in collagens, fibronectin, nidogen, laminin, hyaluronic acid, and proteoglycans. We propose that high antimicrobial activity of AM patches could be attributed to the high retention of gentamicin in this preparation, which could be enabled by the unique ultrastructure of AM stroma, presumably by the structure and composition of the extracellular matrix of AM stroma.

The use of AM patches as vehicles for drug delivery of different antibiotics has been also demonstrated by other studies (Kim et al., 2001; Mencucci et al., 2006; Resch et al., 2011; Yelchuri et al., 2017; Sara et al., 2019). Kim et al. (2001) and Mencucci et al. (2006) have shown that the antibiotic uptake (ofloxacin and netilmicin, respectively) is dose-dependent, it occurs rapidly, and the antimicrobial activity of the antibiotic-treated AM is still present up to 6 h (Kim et al., 2001) or even 6 days (Mencucci et al., 2006) after treatment. Moreover, the use of AM patches for drug delivery has been demonstrated also by Resch et al. (2011), who have shown that AM patches can be loaded with ofloxacin, and allow slow release for up to 7 h. Similarly, Yelchuri et al. (2017) have shown that AM patches are a suitable vehicle for drug delivery of moxifloxacin, which can be detected in AM up to 7 weeks after uptake and the same research group has also shown that AM patches could be used as a carrier for extended-release of cefazolin as they allow the release of the antibiotic for up to 5 days after uptake (Yelchuri et al., 2017; Sara et al., 2019).

Importantly, Aykut et al. (2015) have shown that high concentrations of antimicrobials (penicillin, 50 mg/ml, streptomycin 50 mg/ml, neomycin 100 mg/ml, amphotericin B 2.5 mg/ml) are toxic to AM and may eliminate other beneficial properties of AM. Therefore, it is important to evaluate the effect of the drugs used, to avoid eliminating other beneficial properties, such as anti-fibrotic activity, promotion of epithelization, etc.

The use of AM patches in reconstructive and regenerative urology has been investigated by our and other research groups. Jerman et al. have shown that AM patches enable the development of tissue-engineered urothelium with molecular and ultrastructural properties comparable to that of native urothelium (Jerman et al., 2014). Moreover, using *in vivo* models researchers have shown that AM patches have potential for treatment of ureteral injuries, bladder augmentation and urethroplasty (Iijima et al., 2007; Shakeri et al., 2008, 2009; Wang et al., 2014; Adamowicz et al., 2016; Barski et al., 2017). Koziak et al. even performed two studies using AM patches for reconstructive surgery of the ureteral obstruction in patients, which showed promising results (Koziak et al., 2004, 2007). To conclude, since these surgical procedures include the use of antibiotics, we believe that antibiotic-impregnated AM patches would offer a beneficial approach, since AM would not only enable a better regeneration, but would also represent a topical application of the antibiotic to prevent post-operative infections.

### Fresh and Cryopreserved AM Homogenates Have a Powerful Antimicrobial Effect

To compare the antimicrobial effect of different AM preparations, we prepared homogenates of fAM and cAM and performed antimicrobial efficiency assays. Homogenates of fAM and cAM had a potent antimicrobial effect on all tested Gram-positive and Gram-negative strains, with the exception of *S. marcescens*. Our results showed that different protocols for the preparation and storage of AM homogenates evoke different extent of the antimicrobial effect. Namely, fAM homogenate had the greatest antimicrobial effect on all tested strains. Furthermore, the range of the antimicrobial effect differed between cAM homogenates. In comparison to the antimicrobial effect of fAM homogenate, the range of antimicrobial effect of cAM (10 weeks at −20°C) homogenate decreased for 17%; the range of antimicrobial effect of cAM (1 week at −80°C) homogenate decreased for 21% and the range of antimicrobial effect for cAM (10 weeks at −80°C) homogenate decreased for 36%. These results indicated that the length and the temperature of storage crucially affect the antimicrobial properties of AM and among the preservation methods tested, cryopreservation of AM homogenate at −20°C is evidently the most optimal.

The agar diffusion method and the broth or agar dilution methods are considered the gold-standard clinical antimicrobial susceptibility testing methods (Jorgensen and Ferraro, 2009; Balouiri et al., 2016; Schumacher et al., 2018). In this study, we have demonstrated the antimicrobial efficiency of AM homogenates using the agar diffusion method. While the agar diffusion method offers many advantages over other methods, such as simplicity, low cost, the ability to test vast numbers of microorganisms and antimicrobial agents, the ease to interpret results and more importantly, the good correlation between the *in vitro* data and the *in vivo* evolution, this method still has some limitations. For example, it cannot distinguish between bactericidal and bacteriostatic effects (Balouiri et al., 2016).

However, we have recently published a study (Šket et al., 2019), in which we have shown the antimicrobial effect of AM homogenates using the broth and agar dilution methods. We have used the selected bacterial strains that were also used in this study. Namely, we have evaluated the susceptibility of the clinical strain of uropathogenic *E. coli* (UPEC DL94), the wild-type strain of *S. aureus*, and a wild-type strain and a clinical strain of *S. marcescens* to 12 biological samples of AM homogenates. Our results show that AM homogenate had a bacteriostatic effect on UPEC and *S. aureus*, however, the use of higher dilutions of AM homogenate resulted in a bactericidal effect on both strains. As shown also in this study, *S. marcescens* was resistant to the growth-inhibitory substances of AM homogenate (Šket et al., 2019).

Since AM patches elicited no antimicrobial effect, but the AM homogenate did, we presumed that antimicrobial factors are produced and stored in the amniotic cells and on the components of the extracellular matrix in AM stroma. Nevertheless, some researchers have shown a minimal antimicrobial effect of fresh or cryopreserved AM patches (Kjaergaard et al., 2001; Zare Bidaki et al., 2012; Tehrani et al., 2013, 2017; Parthasarathy et al., 2014), but we believe that the observed effect might have been the result of damage to amniotic epithelial cells during manual separation of AM from chorion, resulting in the release of antimicrobial factors from amniotic cells. In light of our results, which showed that the manner of preparation crucially affects the antimicrobial efficiency of AM, we attribute these discrepancies to the absence of a standardized procedure for AM preparation.

We have shown that AM homogenates have a potent antimicrobial effect on several clinical strains of UPEC. Moreover, we detected a strong antimicrobial effect of AM homogenates on the UPEC DL90 strain, which is otherwise resistant to ampicillin, chloramphenicol, kanamycin, streptomycin, tetracycline, trimethoprim, nalidixic acid, sulfamethoxazole/trimethoprim, metronidazole, cipfrofloxacin and norfloxacin (Supplementary Table 1) (Rijavec et al., 2006; Starčič Erjavec et al., 2007). Similarly, AM homogenates also had a potent antimicrobial effect on the UPEC DL94 (resistant to tetracycline), DL101 (resistant to chloramphenicol, tetracycline, nalidixic acid) and DL102 (resistant to chloramphenicol). Since the antibiotic resistance in uropathogens is increasing, the development of new antimicrobials is even more important, and the AM homogenate might be a good candidate.

### Use of AM in Clinical Practice: Current Status and Future Applications

AM is already being used in the clinic, most commonly in the form of patches, especially in the field of ophthalmology for the treatment of epithelial defects or ulcers (Jirsova and Jones, 2017). In the recent years, there was also an increase in the use of AM for treatment of burns and chronic wounds and also in multiple surgical procedures, for example for prevention of post-operative adhesions (Lo and Pope, 2009; Silini et al., 2015; Castellanos et al., 2017; Barski et al., 2018). Moreover, since AM is regarded as medical waste, its use is ethically acceptable.

Our and other research groups have shown that AM can be used as an extended-release delivery system for antibiotics. The use of AM in this manner has many advantages, such as the simplicity and low-cost of preparation and storage, high retention of antibiotics and also many intrinsic properties of AM (promotion of epithelization, decreased fibrosis, immunomodulatory activity etc.) (Silini et al., 2015; Magatti et al., 2017; Barski et al., 2018) that are especially beneficial in regenerative medicine. On the other hand, AM patches have limited shelf life and the heterogeneity of AM is a challenge, because there is some variability between donors and also between different regions of the same AM (Banerjee et al., 2015; Centurione et al., 2018) (Table 2).

**TABLE 2:** Overview of the advantages and disadvantages of the use of antibiotic-impregnated AM patches and AM homogenate.

Advantages	Disadvantages
**Antibiotic-impregnated AM patches**	
Simple and low-cost preparation and storage	Biological heterogeneity of AM
High retention of antibiotics	Limited storage period
Beneficial intrinsic properties of AM	
Already used in clinic, no ethical concerns	
**AM homogenate**	
Simple and low cost preparation and storage	Limited storage period
Broad-spectrum antimicrobial efficiency	The possibility of development of bacterial resistance to AM homogenate
Beneficial intrinsic properties of AM	Biological heterogeneity of AM

In this study, we show encouraging results regarding the broad-spectrum antimicrobial activity of fAM and cAM homogenates. Similarly to AM patches, the use of AM homogenate has several advantages, such as the aforementioned simplicity and low-cost of preparation and storage, broad-spectrum antimicrobial activity and beneficial intrinsic properties of AM. Possible challenges of the use of AM homogenate include limited shelf life, the possibility of development of bacterial resistance to AM homogenate and biological heterogeneity of AM (Table 2).

The next step is an *in vivo* study, in which fAM and cAM homogenates will be applied as intravesical therapy, especially to combat recurrent UTIs and infections with multiple-resistant bacteria. Since the use of intravesical antibiotics has been shown to have a greater effect on bacteria at a local level while reducing systemic absorption and their associated side effects (Pietropaolo et al., 2018), we believe that the intravesical application of AM homogenates would be most beneficial. AM homogenates could be deemed as multitarget therapy, as there are multiple components of the AM homogenates, such as α- and β-defensins, SLPI and elafin, that act as antimicrobial molecules. Furthermore, AM also has numerous properties which make it suitable for clinical use, e.g., low immunogenicity (Kubo et al., 2001; Szekeres-Bartho, 2002), anti-inflammatory (Kronsteiner et al., 2011; Li et al., 2015; Magatti et al., 2015, 2016; Pianta et al., 2015) and antifibrotic activity (Tseng et al., 1999; Kim et al., 2000; Sant’Anna et al., 2011), angiogenic and anti-angiogenic activity (Kim and Tseng, 1995; Hao et al., 2000; Paeini-Vayghan et al., 2011; Niknejad et al., 2013) and promotion of epithelization (Fukuda et al., 1999; Kim et al., 2000; Nakamura et al., 2004; Jin et al., 2015). Moreover, it has been shown by our research group and others that AM is also beneficial for the regeneration of urothelium (Sharifiaghdas et al., 2007; Jerman et al., 2014; Adamowicz et al., 2016). Therefore, studies suggest that AM homogenates would not only contribute to treatment as antimicrobial agents but at the same time also promote the regeneration of the urothelium (Table 2).

Another advantage for use of AM in regenerative medicine is also its biological origin since biological grafts are less prone to infections than synthetic grafts (Brennan et al., 2006; FitzGerald and Kumar, 2014). There is a need to develop new therapeutic approaches for the treatment of UTIs, first due to the emergence and spread of antibiotic-resistant uropathogenic bacteria and also due to the detrimental effect that certain antibiotics have on epithelial cells. For example, floroquinolones induce S-phase arrest or S/G2 phase arrest and apoptosis (Yadav and Talwar, 2019). Furthermore, since chronic UTIs have also been shown that in some cases increase the risk of development of bladder cancer (Abol-Enein, 2008; Anderson-Otunu and Akhtar, 2016; Bayne et al., 2018), there is a great need to develop novel antimicrobial agents for the treatment of UTIs.

To sum up, antimicrobial activity is another very beneficial property of AM and our study showed the importance of choosing the correct preservation procedure to ensure the maintenance of the intrinsic antimicrobial efficacy of AM. As we have shown that AM homogenates have a potent antimicrobial effect on several clinical UPEC strains, further research to explore the use of AM in treating UTIs *in vivo* is needed, to provide the final breakthrough before using AM as a new antimicrobial agent.

## Data Availability Statement

All datasets generated for this study are included in the article/Supplementary Material.

## Ethics Statement

The studies involving human participants were reviewed and approved by the study on human amniotic membrane was approved by the National Medical Ethics Committee of Republic of Slovenia. The patients/participants provided their written informed consent to participate in this study.

## Author Contributions

TR, MS, and MK designed the study, reviewed and edited the manuscript, and approved the final manuscript. TR and MK performed the experiments and interpreted the results. TR prepared the original manuscript.

## Conflict of Interest

The authors declare that the research was conducted in the absence of any commercial or financial relationships that could be construed as a potential conflict of interest.

## Abbreviations

AM: amniotic membrane
cAM: cryopreserved amniotic membrane
cAM + atb: antibiotic-impregnated cryopreserved amniotic membrane
fAM: fresh amniotic membrane
UPEC: uropathogenic *Escherichia coli;*
Gm^*r*^: gentamicin-resistant.
