# Supplementary material for: Amniotic Membrane Preparation Crucially Affects Its Broad-Spectrum Activity Against Uropathogenic Bacteria
Source: Front Microbiol. 2020 Mar 24;11:469. doi: 10.3389/fmicb.2020.00469 (PMC7107013; doi:10.3389/fmicb.2020.00469)
Supplement: Supplementary file 1 [file Data_Sheet_1.docx]

Supplementary Material

Amniotic membrane preparation crucially affects its broad-spectrum activity against uropathogenic bacteria

Taja Železnik Ramuta^1^, Marjanca Starčič Erjavec^2^, Mateja Erdani Kreft^1^*

^1^ Institute of Cell Biology, Faculty of Medicine, University of Ljubljana, Ljubljana, Slovenia

^2^ Department of Biology, Biotechnical Faculty, University of Ljubljana, Ljubljana, Slovenia

**Supplementary Table 1: Characteristics of UPEC DL strains isolated from human samples.** The presence of virulence-associated genes in each DL strain is marked (+) for presence of selected genes and (-) for absence of selected genes. DL strains that are resistant to selected antibiotics are marked with (+) and strains that are susceptible to selected antibiotics are marked with (-). n/a – not applicable.

|  |  | **DL strain** | | | | |
| --- | --- | --- | --- | --- | --- | --- |
|  |  | **DL88** | **DL90** | **DL94** | **DL101** | **DL102** |
|  | Serotype | O13:H4 | Ont:H- | O21:H- | O23:H31 | Orough:H- |
|  | Phylogenetic group | B2 | A | A | B1 | B2 |
|  | Phylogenetic subgroup | B2_3_ | A1 | A1 | n/a | B2_3_ |
| **Virulence-associated genes** | *cnf* | - | - | - | - | + |
|  | *hlyA* | - | - | - | - | + |
|  | *usp* | + | + | - | - | + |
|  | *ibeA* | - | - | - | - | + |
|  | *fimH* | + | + | + | + | + |
|  | *papC* | - | + | + | - | + |
|  | *papGII* | - | - | + | - | - |
|  | *pap GIII* | - | - | - | - | + |
|  | *sfa* | - | - | - | - | + |
|  | *afa/draBC* | - | - | - | - | - |
|  | *iucD* | - | - | - | - | - |
|  | *iroCD* | - | - | - | - | + |
|  | *iroN* | - | - | - | - | + |
|  | *fyuA* | + | - | + | + | + |
|  | *ireA* | - | + | - | - | + |
|  | *traT* | - | + | - | - | - |
| **Antibiotic resistance** | Ampicillin | - | + | - | - | - |
|  | Chloramphenicol | - | + | - | + | + |
|  | Kanamycin | - | + | - | - | - |
|  | Streptomycin | - | + | - | - | - |
|  | Tetracycline | - | + | + | + | - |
|  | Trimethoprim | - | + | - | - | - |
|  | Nalidixic acid | - | + | - | + | - |
|  | Spectinomycin | - | - | - | - | - |
|  | Sulfamethoxazole/ Trimethoprim | - | + | - | - | - |
|  | Metronidazole | - | + | - | - | - |
|  | Ciprofloxacin | - | + | - | - | - |
|  | Norfloxacin | - | + | - | - | - |

**Supplementary Table 2: Homogenates of fresh (fAM) and cryopreserved amniotic membrane (cAM) have powerful antimicrobial effect on all tested strains, except on *S. marcescens***. **Mean diameters ± standard errors (mm) of the antimicrobial zones due to the antimicrobial effect of fAM and cAM homogenates on tested strains.** The mean diameters and standard errors were calculated from 3–5 biological repeats of AM and 6–30 technical repeats for each strain for each assay. Larger volumes of homogenates (10 µl) have greater antimicrobial effect than smaller volumes (5 µl). (–) No antimicrobial effect. The *E. coli* DH5α strain was used as a control strain.

|  | **AM homogenate** | | | | | | | |
| --- | --- | --- | --- | --- | --- | --- | --- | --- |
|  | **(mean diameter of the antimicrobial zone ± standard error (mm))** | | | | | | | |
|  | **fAM** | | **cAM (1 week at** –**80°C)** | | **cAM (10 weeks at** –**80°C)** | | **cAM (10 weeks at** –**20°C)** | |
|  | **5 µl** | **10 µl** | **5 µl** | **10 µl** | **5 µl** | **10 µl** | **5 µl** | **10 µl** |
| **Gram-positive tested strains** | | | | | | | | |
| ***S. aureus*** | 16.0 ± 0.2 | 19.0 ± 0.3 | 12.3 ± 0.4 | 16.1 ± 0.4 | 10.2 ± 0.6 | 14.5 ± 0.3 | 14.3 ± 0.5 | 17.7 ± 0.4 |
|  | 3 independent replications of experiments using 3 biological samples (6 technical repeats were performed for each biological sample) | | 3 independent replications of experiments using 3 biological samples (3-6 technical repeats were performed for each biological sample) | | 5 independent replications of experiments using 5 biological samples (5-6 technical repeats were performed for each biological sample) | | 3 independent replications of experiments using 3 biological samples (3-6 technical repeats were performed for each biological sample) | |
| ***S. saprophyticus*** | 8.2 ± 0.4 | 10.2 ± 0.4 | 6.1 ± 0.3 | 8.5 ± 0.6 | 4.3 ± 0.5 | 8.1 ± 0.5 | 6.8 ± 0.7 | 9.3 ± 0.3 |
|  | 3 independent replications of experiments using 3 biological samples (6 technical repeats were performed for each biological sample) | | 3 independent replications of experiments using 3 biological samples (3-6 technical repeats were performed for each biological sample) | | 5 independent replications of experiments using 5 biological samples (6 technical repeats were performed for each biological sample) | | 3 independent replications of experiments using 3 biological samples (3-6 technical repeats were performed for each biological sample) | |
| **Gram-negative tested strains** | | | | | | | | |
| **UPEC DL94** | 7.6 ± 0.2 | 9.5 ± 0.2 | 5.3 ± 0.2 | 7.7 ± 0.3 | 5.5 ± 0.2 | 7.0 ± 0.2 | 5.7 ± 0.4 | 8.5 ± 0.2 |
|  | 3 independent replications of experiments using 3 biological samples (6 technical repeats were performed for each biological sample) | | 3 independent replications of experiments using 3 biological samples (3-6 technical repeats were performed for each biological sample) | | 5 independent replications of experiments using 5 biological samples (6 technical repeats were performed for each biological sample) | | 3 independent replications of experiments using 3 biological samples (3-6 technical repeats were performed for each biological sample) | |
| ***E. coli* DH5α** | 6.1 ± 0.2 | 8.2 ± 0.2 | 5.3 ± 0.2 | 7.2 ± 0.2 | 3.6 ± 0.4 | 5.4 ± 0.4 | 4.8 ± 0.2 | 7.1 ± 0.1 |
|  | 3 independent replications of experiments using 3 biological samples (6 technical repeats were performed for each biological sample) | | 3 independent replications of experiments using 3 biological samples (3-6 technical repeats were performed for each biological sample) | | 5 independent replications of experiments using 5 biological samples (5-6 technical repeats were performed for each biological sample) | | 3 independent replications of experiments using 3 biological samples (3-6 technical repeats were performed for each biological sample) | |
| ***M. morganii*** | 6.3 ± 0.1 | 8.1 ± 0.2 | 4.9 ± 0.1 | 6.9 ± 0.2 | 3.7 ± 0.3 | 5.9 ± 0.2 | 5.0 ± 0.5 | 7.2 ± 0.1 |
|  | 3 independent replications of experiments using 3 biological samples (6 technical repeats were performed for each biological sample) | | 3 independent replications of experiments using 3 biological samples (3-6 technical repeats were performed for each biological sample) | | 5 independent replications of experiments using 5 biological samples (5-6 technical repeats were performed for each biological sample) | | 3 independent replications of experiments using 3 biological samples (3-6 technical repeats were performed for each biological sample) | |
| ***P. rettgeri*** | 6.1 ± 0.2 | 7.8 ± 0.2 | 4.5 ± 0.3 | 6.4 ± 0.1 | 3.1 ± 0.3 | 4.9 ± 0.4 | 5.2 ± 0.4 | 7.0 ± 0.1 |
|  | 3 independent replications of experiments using 3 biological samples (6 technical repeats were performed for each biological sample) | | 3 independent replications of experiments using 3 biological samples (3-6 technical repeats were performed for each biological sample) | | 5 independent replications of experiments using 5 biological samples (6 technical repeats were performed for each biological sample) | | 3 independent replications of experiments using 3 biological samples (3-6 technical repeats were performed for each biological sample) | |
| **UPEC DL102** | 6.1 ± 0.1 | 7.8 ± 0.2 | 4.9 ± 0.2 | 6.8 ± 0.2 | 4.4 ± 0.1 | 6.0 ± 0.2 | 3.8 ± 0.3 | 6.9 ± 0.1 |
|  | 3 independent replications of experiments using 3 biological samples (6 technical repeats were performed for each biological sample) | | 3 independent replications of experiments using 3 biological samples (3-6 technical repeats were performed for each biological sample) | | 5 independent replications of experiments using 5 biological samples (6 technical repeats were performed for each biological sample) | | 3 independent replications of experiments using 3 biological samples (3-6 technical repeats were performed for each biological sample) | |
| **UPEC DL90** | 5.8 ± 0.2 | 7.6 ± 0.1 | 3.4 ± 0.2 | 5.3 ± 0.3 | 1.8 ± 0.3 | 4.1 ± 0.3 | 4.3 ± 0.3 | 6.0 ± 0.2 |
|  | 3 independent replications of experiments using 3 biological samples (6 technical repeats were performed for each biological sample) | | 3 independent replications of experiments using 3 biological samples (3-6 technical repeats were performed for each biological sample) | | 5 independent replications of experiments using 5 biological samples (6 technical repeats were performed for each biological sample) | | 3 independent replications of experiments using 3 biological samples (3-6 technical repeats were performed for each biological sample) | |
| **UPEC DL101** | 6.5 ± 0.3 | 7.6 ± 0.2 | 4.6 ± 0.1 | 6.1 ± 0.2 | 3.2 ± 0.3 | 5.3 ± 0.3 | 4.5 ± 0.2 | 6.0 ± 0.2 |
|  | 3 independent replications of experiments using 3 biological samples (6 technical repeats were performed for each biological sample) | | 3 independent replications of experiments using 3 biological samples (3-6 technical repeats were performed for each biological sample) | | 5 independent replications of experiments using 5 biological samples (6 technical repeats were performed for each biological sample) | | 3 independent replications of experiments using 3 biological samples (3-6 technical repeats were performed for each biological sample) | |
| ***P. mirabilis*** | 5.4 ± 0.2 | 7.5 ± 0.2 | 4.3 ± 0.2 | 6.4 ± 0.1 | 3.1 ± 0.3 | 5.4 ± 0.2 | 3.5 ± 0.5 | 6.5 ± 0.1 |
|  | 3 independent replications of experiments using 3 biological samples (6 technical repeats were performed for each biological sample) | | 3 independent replications of experiments using 3 biological samples (3-6 technical repeats were performed for each biological sample) | | 5 independent replications of experiments using 5 biological samples (6 technical repeats were performed for each biological sample) | | 3 independent replications of experiments using 3 biological samples (3-6 technical repeats were performed for each biological sample) | |
| **UPEC DL88** | 6.0 ± 0.2 | 7.3 ± 0.2 | 4.6 ± 0.1 | 6.5 ± 0.1 | 3.7 ± 0.3 | 5.3 ± 0.4 | 4.5 ± 0.4 | 6.7 ± 0.2 |
|  | 3 independent replications of experiments using 3 biological samples (6 technical repeats were performed for each biological sample) | | 3 independent replications of experiments using 3 biological samples (3-6 technical repeats were performed for each biological sample) | | 5 independent replications of experiments using 5 biological samples (5-6 technical repeats were performed for each biological sample) | | 3 independent replications of experiments using 3 biological samples (3-6 technical repeats were performed for each biological sample) | |
| ***Enterobacter spp.*** | 4.9 ± 0.2 | 7.3 ± 0.2 | 3.5 ± 0.2 | 5.1 ± 0.3 | 2.1 ± 0.2 | 3.8 ± 0.3 | 2.2 ± 0.4 | 5.3 ± 0.2 |
|  | 3 independent replications of experiments using 3 biological samples (6 technical repeats were performed for each biological sample) | | 3 independent replications of experiments using 3 biological samples (3-6 technical repeats were performed for each biological sample) | | 5 independent replications of experiments using 5 biological samples (6 technical repeats were performed for each biological sample) | | 3 independent replications of experiments using 3 biological samples (3-6 technical repeats were performed for each biological sample) | |
| ***K. pneumoniae*** | 5.6 ± 0.1 | 7.1 ± 0.2 | 4.3 ± 0.1 | 6.4 ± 0.2 | 3.5 ± 0.3 | 5.6 ± 0.3 | 4.7 ± 0.2 | 7.0 ± 0.1 |
|  | 3 independent replications of experiments using 3 biological samples (6 technical repeats were performed for each biological sample) | | 3 independent replications of experiments using 3 biological samples (3-6 technical repeats were performed for each biological sample) | | 5 independent replications of experiments using 5 biological samples (5-6 technical repeats were performed for each biological sample) | | 3 independent replications of experiments using 3 biological samples (3-6 technical repeats were performed for each biological sample) | |
| ***S. marcescens*** | – | – | – | – | – | – | – | – |
|  | 3 independent replications of experiments using 3 biological samples (6 technical repeats were performed for each biological sample) | | 3 independent replications of experiments using 3 biological samples (3-6 technical repeats were performed for each biological sample) | | 5 independent replications of experiments using 5 biological samples (6 technical repeats were performed for each biological sample) | | 3 independent replications of experiments using 3 biological samples (3-6 technical repeats were performed for each biological sample) | |
| **Average antimicrobial zone for all susceptible strains** | **7.0 ± 0.2** | **8.8 ± 0.2** | **5.2 ± 0.2** | **7.3 ± 0.2** | **4.0 ± 0.3** | **6.3 ± 0.3** | **5.3 ± 0.4** | **7.8 ± 0.2** |

**Supplementary Table 3: The analysis of variance (ANOVA) of the antimicrobial effect of AM homogenates for each tested strain**. ANOVA was performed to compare the ranges of the antimicrobial zones, caused by fAM, cAM (1 week at –80°C), cAM (10 weeks at –80°C) and cAM (10 weeks at –20°C) homogenates for each tested strain separately; namely, 5 µl and 10 µl of AM homogenates were used. p<0.05 values were considered statistically significant. N/A – not applicable. DF – degrees of freedom.

|  | ***S. aureus*** | | | | ***S. saprophyticus*** | | | | **UPEC DL94** | | | | ***E. coli* DH5α** | | | | ***M. morganii*** | | | |
| --- | --- | --- | --- | --- | --- | --- | --- | --- | --- | --- | --- | --- | --- | --- | --- | --- | --- | --- | --- | --- |
| **Volume of AM homogenate used** | **5 µl** | | **10 µl** | | **5 µl** | | **10 µl** | | **5 µl** | | **10 µl** | | **5 µl** | | **10 µl** | | **5 µl** | | **10 µl** | |
| ***Tukey's multiple comparisons test*** | **Adjusted P Value** | **DF** | **Adjusted P Value** | **DF** | **Adjusted P Value** | **DF** | **Adjusted P Value** | **DF** | **Adjusted P Value** | **DF** | **Adjusted P Value** | **DF** | **Adjusted P Value** | **DF** | **Adjusted P Value** | **DF** | **Adjusted P Value** | **DF** | **Adjusted P Value** | **DF** |
| **fAM vs. cAM (1 week at  –80°C)** | < 0,0001 | 68 | < 0,0001 | 68 | 0,0317 | 68 | 0,2526 | 68 | < 0,0001 | 68 | < 0,0001 | 68 | 0,4272 | 68 | 0,3891 | 67 | 0,0011 | 68 | 0,0029 | 67 |
| **fAM vs. cAM (10 weeks at –80°C)** | < 0,0001 | 68 | < 0,0001 | 68 | < 0,0001 | 68 | 0,0259 | 68 | < 0,0001 | 68 | < 0,0001 | 68 | < 0,0001 | 68 | < 0,0001 | 67 | < 0,0001 | 68 | < 0,0001 | 67 |
| **fAM vs. cAM (10 weeks at –20°C)** | 0,4369 | 68 | 0,1755 | 68 | 0,5815 | 68 | 0,7874 | 68 | 0,0001 | 68 | 0,0403 | 68 | 0,3333 | 68 | 0,3182 | 67 | 0,0585 | 68 | 0,0256 | 67 |
| **cAM (1 week at –80°C) vs. cAM (10 weeks at  –80°C)** | 0,0086 | 68 | 0,0112 | 68 | 0,0546 | 68 | 0,9614 | 68 | 0,8894 | 68 | 0,2062 | 68 | 0,002 | 68 | 0,015 | 67 | 0,0019 | 68 | 0,0114 | 67 |
| **cAM (1 week at –80°C) vs. cAM (10 weeks at  –20°C)** | 0,2413 | 68 | 0,0692 | 68 | 0,8911 | 68 | 0,8296 | 68 | 0,7858 | 68 | 0,1736 | 68 | 0,9263 | 68 | 0,9994 | 67 | 0,996 | 68 | 0,9044 | 67 |
| **cAM (10 weeks at -80°C) vs. cAM (10 weeks at -20°C)** | 0,0008 | 68 | < 0,0001 | 68 | 0,0714 | 68 | 0,4396 | 68 | 0,9571 | 68 | 0,0002 | 68 | 0,2577 | 68 | 0,0223 | 67 | 0,0369 | 68 | 0,0009 | 67 |
| **F value** | 23,13 | | 29,26 | | 11,42 | | 3,03 | | 26,6 | | 25,63 | | 11,09 | | 11,01 | | 22,7 | | 22,86 | |
|  | ***P. rettgeri*** | | | | **UPEC DL102** | | | | **UPEC DL90** | | | | **UPEC DL101** | | | | ***P. mirabilis*** | | | |
| **Volume of AM homogenate used** | **5 µl** | | **10 µl** | | **5 µl** | | **10 µl** | | **5 µl** | | **10 µl** | | **5 µl** | | **10 µl** | | **5 µl** | | **10 µl** | |
| ***Tukey's multiple comparisons test*** | **Adjusted P Value** | DF | **Adjusted P Value** | DF | **Adjusted P Value** | DF | **Adjusted P Value** | DF | **Adjusted P Value** | DF | **Adjusted P Value** | DF | **Adjusted P Value** | DF | **Adjusted P Value** | DF | **Adjusted P Value** | DF | **Adjusted P Value** | DF |
| **fAM vs. cAM (1 week at  –80°C)** | 0,0066 | 68 | 0,059 | 68 | < 0,0001 | 68 | 0,0265 | 68 | < 0,0001 | 68 | < 0,0001 | 68 | 0,0008 | 68 | 0,0042 | 68 | 0,0496 | 68 | 0,0063 | 67 |
| **fAM vs. cAM (10 weeks at –80°C)** | < 0,0001 | 68 | < 0,0001 | 68 | < 0,0001 | 68 | < 0,0001 | 68 | < 0,0001 | 68 | < 0,0001 | 68 | < 0,0001 | 68 | < 0,0001 | 68 | < 0,0001 | 68 | < 0,0001 | 67 |
| **fAM vs. cAM (10 weeks at –20°C)** | 0,5417 | 68 | 0,4377 | 68 | < 0,0001 | 68 | 0,0839 | 68 | 0,0323 | 68 | 0,0112 | 68 | 0,0184 | 68 | 0,0023 | 68 | 0,0091 | 68 | 0,0135 | 67 |
| **cAM (1 week at –80°C) vs. cAM (10 weeks at  –80°C)** | 0,0071 | 68 | 0,0233 | 68 | 0,0688 | 68 | 0,1094 | 68 | < 0,0001 | 68 | 0,0687 | 68 | 0,0053 | 68 | 0,1825 | 68 | 0,0089 | 68 | 0,0042 | 67 |
| **cAM (1 week at –80°C) vs. cAM (10 weeks at  –20°C)** | 0,7231 | 68 | 0,77 | 68 | 0,0111 | 68 | 0,9737 | 68 | 0,3178 | 68 | 0,5003 | 68 | 0,9983 | 68 | 0,9978 | 68 | 0,5046 | 68 | 0,9952 | 67 |
| **cAM (10 weeks at  –80°C) vs. cAM (10 weeks at  –20°C)** | 0,0074 | 68 | 0,0007 | 68 | 0,3302 | 68 | 0,0325 | 68 | < 0,0001 | 68 | 0,0005 | 68 | 0,157 | 68 | 0,2702 | 68 | 0,8937 | 68 | 0,0017 | 67 |
| **F value** | 17,82 | | 15,82 | | 28,37 | | 13,16 | | 50,5 | | 26,5 | | 21,16 | | 15,2 | | 13,62 | | 23,18 | |
|  | **UPEC DL88** | | | | ***Enterobacter sp.*** | | | | ***K. pneumoniae*** | | | | **UPEC DL88-Gm^r^** | | | | **UPEC DL90-Gm^r^** | | | |
| **Volume of AM homogenate used** | **5 µl** | | **10 µl** | | **5 µl** | | **10 µl** | | **5 µl** | | **10 µl** | | **5 µl** | | **10 µl** | | **5 µl** | | **10 µl** | |
| ***Tukey's multiple comparisons test*** | **Adjusted P Value** | DF | **Adjusted P Value** | DF | **Adjusted P Value** | DF | **Adjusted P Value** | DF | **Adjusted P Value** | DF | **Adjusted P Value** | DF | **Adjusted P Value** | DF | **Adjusted P Value** | DF | **Adjusted P Value** | DF | **Adjusted P Value** | DF |
| **fAM vs. cAM (1 week at  –80°C)** | 0,0079 | 68 | 0,3683 | 67 | 0,0007 | 68 | 0,0001 | 68 | 0,0028 | 68 | 0,3597 | 65 | < 0,0001 | 39 | < 0,0001 | 36 | < 0,0001 | 39 | < 0,0001 | 36 |
| **fAM vs. cAM (10 weeks at –80°C)** | < 0,0001 | 68 | < 0,0001 | 67 | < 0,0001 | 68 | < 0,0001 | 68 | < 0,0001 | 68 | 0,0001 | 65 | < 0,0001 | 39 | < 0,0001 | 36 | < 0,0001 | 39 | < 0,0001 | 36 |
| **fAM vs. cAM (10 weeks at –20°C)** | 0,0803 | 68 | 0,563 | 67 | < 0,0001 | 68 | 0,0007 | 68 | 0,2349 | 68 | 0,9989 | 65 | N/A |  | N/A |  | N/A |  | N/A |  |
| **cAM (1 week at –80°C) vs. cAM (10 weeks at  –80°C)** | 0,135 | 68 | 0,0645 | 67 | 0,0003 | 68 | 0,0227 | 68 | 0,0607 | 68 | 0,1127 | 65 | 0,0003 | 39 | 0,166 | 36 | 0,0008 | 39 | 0,0221 | 36 |
| **cAM (1 week at –80°C) vs. cAM (10 weeks at  –20°C)** | 0,9997 | 68 | 0,9907 | 67 | 0,0486 | 68 | 0,966 | 68 | 0,907 | 68 | 0,5205 | 65 | N/A |  | N/A |  | N/A |  | N/A |  |
| **cAM (10 weeks at  –80°C) vs. cAM (10 weeks at  –20°C)** | 0,5235 | 68 | 0,0265 | 67 | 0,9999 | 68 | 0,0045 | 68 | 0,0844 | 68 | 0,0013 | 65 | N/A |  | N/A |  | N/A |  | N/A |  |
| **F value** | 11,61 | | 8,794 | | 28,25 | | 27,84 | | 14,87 | | 9,101 | | 84,29 | | 41,34 | | 87,83 | | 57,5 | |

**Supplementary Table 4: The analysis of variance (ANOVA) of the antimicrobial effect of AM homogenates for all tested strains**. ANOVA was performed to compare the ranges of the antimicrobial zones, caused by fAM, cAM (1 week at –80°C), cAM (10 weeks at –80°C) and cAM (10 weeks at –20°C) homogenates for all tested strains; namely, 5 µl and 10 µl of AM homogenates were used. p<0.05 values were considered statistically significant. DF – degrees of freedom.

| **Tukey's multiple comparisons test** | **fAM** | | | | **cAM (1 week at –80°C)** | | | | **cAM (10 weeks at –80°C)** | | | | **cAM (10 weeks at –20°C)** | | | |
| --- | --- | --- | --- | --- | --- | --- | --- | --- | --- | --- | --- | --- | --- | --- | --- | --- |
|  | 5 µl | | 10 µl | | 5 µl | | 10 µl | | 5 µl | | 10 µl | | 5 µl | | 10 µl | |
|  | Adjusted P Value | DF | Adjusted P Value | DF | Adjusted P Value | DF | Adjusted P Value | DF | Adjusted P Value | DF | Adjusted P Value | DF | Adjusted P Value | DF | Adjusted P Value | DF |
| ***E. coli* DH5*a* vs. UPEC88** | > 0.9999 | 235 | 0.3041 | 235 | 0.5777 | 241 | 0.9114 | 157 | > 0.9999 | 406 | > 0.9999 | 399 | > 0.9999 | 70 | 0.9967 | 154 |
| ***E. coli* DH5*a* vs. UPEC90** | 0.9995 | 235 | 0.7925 | 235 | < 0.0001 | 241 | 0.0002 | 157 | 0.0143 | 406 | 0.2363 | 399 | 0.9998 | 70 | 0.1483 | 154 |
| ***E. coli* DH5*a* vs. UPEC94** | < 0.0001 | 235 | 0.0023 | 235 | > 0.9999 | 241 | 0.9914 | 157 | 0.0037 | 406 | 0.0224 | 399 | 0.9758 | 70 | 0.0093 | 154 |
| ***E. coli* DH5*a* vs. UPEC101** | 0.9587 | 235 | 0.7925 | 235 | 0.7029 | 241 | 0.2508 | 157 | 0.9999 | 406 | > 0.9999 | 399 | > 0.9999 | 70 | 0.1483 | 154 |
| ***E. coli* DH5*a* vs. UPEC102** | > 0.9999 | 235 | 0.9935 | 235 | 0.9945 | 241 | 0.9985 | 157 | 0.9033 | 406 | 0.983 | 399 | 0.9061 | 70 | > 0.9999 | 154 |
| ***E. coli* DH5*a* vs*. S. marcescens*** | < 0.0001 | 235 | < 0.0001 | 235 | < 0.0001 | 241 | < 0.0001 | 157 | < 0.0001 | 406 | < 0.0001 | 399 | < 0.0001 | 70 | < 0.0001 | 154 |
| ***E. coli* DH5*a* vs. *P. mirabilis*** | 0.6884 | 235 | 0.6766 | 235 | 0.1566 | 241 | 0.8137 | 157 | 0.9992 | 406 | > 0.9999 | 399 | 0.5767 | 70 | 0.9389 | 154 |
| ***E. coli* DH5*a* vs. *K. pneumoniae*** | 0.9587 | 235 | 0.0296 | 235 | 0.1566 | 241 | 0.8137 | 157 | > 0.9999 | 406 | > 0.9999 | 399 | > 0.9999 | 70 | > 0.9999 | 154 |
| ***E. coli* DH5*a* vs. *Enterobacter spp.*** | 0.011 | 235 | 0.3041 | 235 | < 0.0001 | 241 | < 0.0001 | 157 | 0.1088 | 406 | 0.0239 | 399 | 0.0016 | 70 | 0.0002 | 154 |
| ***E. coli* DH5*a* vs. *S. saprophyticus*** | < 0.0001 | 235 | < 0.0001 | 235 | 0.4503 | 241 | 0.0497 | 157 | 0.9643 | 406 | < 0.0001 | 399 | 0.0586 | 70 | < 0.0001 | 154 |
| ***E. coli* DH5*a* vs. *S. aureus*** | < 0.0001 | 235 | < 0.0001 | 235 | < 0.0001 | 241 | < 0.0001 | 157 | < 0.0001 | 406 | < 0.0001 | 399 | < 0.0001 | 70 | < 0.0001 | 154 |
| ***E. coli* DH5*a* vs. *P. rettgeri*** | > 0.9999 | 235 | 0.9986 | 235 | 0.3897 | 241 | 0.8137 | 157 | 0.9984 | 406 | 0.9993 | 399 | > 0.9999 | 70 | > 0.9999 | 154 |
| ***E. coli* DH5*a* vs. *M. morganii*** | > 0.9999 | 235 | > 0.9999 | 235 | 0.9945 | 241 | > 0.9999 | 157 | > 0.9999 | 406 | 0.9946 | 399 | > 0.9999 | 70 | > 0.9999 | 154 |
| **UPEC88 vs. UPEC90** | > 0.9999 | 235 | > 0.9999 | 235 | 0.0361 | 241 | 0.0906 | 157 | 0.0049 | 406 | 0.3241 | 399 | > 0.9999 | 70 | 0.85 | 154 |
| **UPEC88 vs. UPEC94** | < 0.0001 | 235 | < 0.0001 | 235 | 0.5777 | 241 | 0.1557 | 157 | 0.011 | 406 | 0.0131 | 399 | 0.7668 | 70 | < 0.0001 | 154 |
| **UPEC88 vs. UPEC101** | 0.9021 | 235 | > 0.9999 | 235 | > 0.9999 | 241 | 0.9985 | 157 | 0.9969 | 406 | > 0.9999 | 399 | > 0.9999 | 70 | 0.85 | 154 |
| **UPEC88 vs. UPEC102** | > 0.9999 | 235 | 0.9783 | 235 | 0.9988 | 241 | > 0.9999 | 157 | 0.9761 | 406 | 0.9591 | 399 | 0.9967 | 70 | > 0.9999 | 154 |
| **UPEC88 vs. *S. marcescens*** | < 0.0001 | 235 | < 0.0001 | 235 | < 0.0001 | 241 | < 0.0001 | 157 | < 0.0001 | 406 | < 0.0001 | 399 | < 0.0001 | 70 | < 0.0001 | 154 |
| **UPEC88 vs. *P. mirabilis*** | 0.8107 | 235 | > 0.9999 | 235 | > 0.9999 | 241 | > 0.9999 | 157 | 0.9905 | 406 | > 0.9999 | 399 | 0.9061 | 70 | > 0.9999 | 154 |
| **UPEC88 vs. *K. pneumoniae*** | 0.9865 | 235 | 0.9998 | 235 | > 0.9999 | 241 | > 0.9999 | 157 | > 0.9999 | 406 | > 0.9999 | 399 | > 0.9999 | 70 | 0.9997 | 154 |
| **UPEC88 vs*. Enterobacter spp*.** | 0.0213 | 235 | > 0.9999 | 235 | 0.0613 | 241 | 0.0258 | 157 | 0.0473 | 406 | 0.0394 | 399 | 0.011 | 70 | 0.0203 | 154 |
| **UPEC88 vs. *S. saprophyticus*** | < 0.0001 | 235 | < 0.0001 | 235 | 0.0003 | 241 | < 0.0001 | 157 | 0.9945 | 406 | < 0.0001 | 399 | 0.011 | 70 | < 0.0001 | 154 |
| **UPEC88 vs. *S. aureus*** | < 0.0001 | 235 | < 0.0001 | 235 | < 0.0001 | 241 | < 0.0001 | 157 | < 0.0001 | 406 | < 0.0001 | 399 | < 0.0001 | 70 | < 0.0001 | 154 |
| **UPEC88 vs. *P. rettgeri*** | > 0.9999 | 235 | 0.9444 | 235 | > 0.9999 | 241 | > 0.9999 | 157 | 0.9846 | 406 | 0.9999 | 399 | 0.9967 | 70 | 0.9997 | 154 |
| **UPEC88 vs. *M. morganii*** | 0.9995 | 235 | 0.3594 | 235 | 0.9988 | 241 | 0.9985 | 157 | > 0.9999 | 406 | 0.9842 | 399 | 0.9998 | 70 | 0.9822 | 154 |
| **UPEC90 vs. UPEC94** | < 0.0001 | 235 | < 0.0001 | 235 | < 0.0001 | 241 | < 0.0001 | 157 | < 0.0001 | 406 | < 0.0001 | 399 | 0.5767 | 70 | < 0.0001 | 154 |
| **UPEC90 vs. UPEC101** | 0.4107 | 235 | > 0.9999 | 235 | 0.0204 | 241 | 0.6789 | 157 | 0.1876 | 406 | 0.3245 | 399 | > 0.9999 | 70 | > 0.9999 | 154 |
| **UPEC90 vs. UPEC102** | 0.9968 | 235 | > 0.9999 | 235 | 0.0007 | 241 | 0.0127 | 157 | < 0.0001 | 406 | 0.0025 | 399 | 0.9998 | 70 | 0.3869 | 154 |
| **UPEC90 vs. *S. marcescens*** | < 0.0001 | 235 | < 0.0001 | 235 | < 0.0001 | 241 | < 0.0001 | 157 | 0.0064 | 406 | < 0.0001 | 399 | < 0.0001 | 70 | < 0.0001 | 154 |
| **UPEC90 vs. *P. mirabilis*** | 0.9968 | 235 | > 0.9999 | 235 | 0.2339 | 241 | 0.1557 | 157 | 0.2587 | 406 | 0.2363 | 399 | 0.9758 | 70 | 0.9822 | 154 |
| **UPEC90 vs. *K. pneumoniae*** | > 0.9999 | 235 | 0.9444 | 235 | 0.2339 | 241 | 0.1557 | 157 | 0.0184 | 406 | 0.083 | 399 | > 0.9999 | 70 | 0.2495 | 154 |
| **UPEC90 vs*. Enterobacter spp*.** | 0.1905 | 235 | > 0.9999 | 235 | > 0.9999 | 241 | > 0.9999 | 157 | > 0.9999 | 406 | > 0.9999 | 399 | 0.0263 | 70 | 0.85 | 154 |
| **UPEC90 vs. *S. saprophyticus*** | < 0.0001 | 235 | < 0.0001 | 235 | < 0.0001 | 241 | < 0.0001 | 157 | < 0.0001 | 406 | < 0.0001 | 399 | 0.0044 | 70 | < 0.0001 | 154 |
| **UPEC90 vs. *S. aureus*** | < 0.0001 | 235 | < 0.0001 | 235 | < 0.0001 | 241 | < 0.0001 | 157 | < 0.0001 | 406 | < 0.0001 | 399 | < 0.0001 | 70 | < 0.0001 | 154 |
| **UPEC90 vs. *P. rettgeri*** | 0.9995 | 235 | 0.9998 | 235 | 0.0787 | 241 | 0.1557 | 157 | 0.3 | 406 | 0.8736 | 399 | 0.9758 | 70 | 0.2495 | 154 |
| **UPEC90 vs. *M. morganii*** | 0.9021 | 235 | 0.8416 | 235 | 0.0007 | 241 | 0.0027 | 157 | 0.0049 | 406 | 0.0054 | 399 | 0.9967 | 70 | 0.0817 | 154 |
| **UPEC94 vs. UPEC101** | 0.0213 | 235 | < 0.0001 | 235 | 0.7029 | 241 | 0.006 | 157 | < 0.0001 | 406 | 0.0107 | 399 | 0.7668 | 70 | < 0.0001 | 154 |
| **UPEC94 vs. UPEC102** | < 0.0001 | 235 | < 0.0001 | 235 | 0.9945 | 241 | 0.5255 | 157 | 0.4954 | 406 | 0.5896 | 399 | 0.1206 | 70 | 0.0017 | 154 |
| **UPEC94 vs. *S. marcescens*** | < 0.0001 | 235 | < 0.0001 | 235 | < 0.0001 | 241 | < 0.0001 | 157 | < 0.0001 | 406 | < 0.0001 | 399 | < 0.0001 | 70 | < 0.0001 | 154 |
| **UPEC94 vs. *P. mirabilis*** | < 0.0001 | 235 | < 0.0001 | 235 | 0.1566 | 241 | 0.0906 | 157 | < 0.0001 | 406 | 0.0224 | 399 | 0.0263 | 70 | < 0.0001 | 154 |
| **UPEC94 vs. *K. pneumoniae*** | < 0.0001 | 235 | < 0.0001 | 235 | 0.1566 | 241 | 0.0906 | 157 | 0.0028 | 406 | 0.1134 | 399 | 0.9061 | 70 | 0.004 | 154 |
| **UPEC94 vs. *Enterobacter spp.*** | < 0.0001 | 235 | < 0.0001 | 235 | < 0.0001 | 241 | < 0.0001 | 157 | < 0.0001 | 406 | < 0.0001 | 399 | < 0.0001 | 70 | < 0.0001 | 154 |
| **UPEC94 vs. *S. saprophyticus*** | 0.5491 | 235 | 0.6766 | 235 | 0.4503 | 241 | 0.6789 | 157 | 0.3447 | 406 | 0.4246 | 399 | 0.7668 | 70 | 0.5497 | 154 |
| **UPEC94 vs. *S. aureus*** | < 0.0001 | 235 | < 0.0001 | 235 | < 0.0001 | 241 | < 0.0001 | 157 | < 0.0001 | 406 | < 0.0001 | 399 | < 0.0001 | 70 | < 0.0001 | 154 |
| **UPEC94 vs. *P. rettgeri*** | < 0.0001 | 235 | < 0.0001 | 235 | 0.3897 | 241 | 0.0906 | 157 | < 0.0001 | 406 | 0.0004 | 399 | 0.9998 | 70 | 0.004 | 154 |
| **UPEC94 vs. *M. morganii*** | 0.0012 | 235 | 0.0016 | 235 | 0.9945 | 241 | 0.8137 | 157 | 0.011 | 406 | 0.4897 | 399 | 0.9967 | 70 | 0.0203 | 154 |
| **UPEC101 vs. UPEC102** | 0.9865 | 235 | > 0.9999 | 235 | 0.9998 | 241 | 0.9114 | 157 | 0.3447 | 406 | 0.9509 | 399 | 0.9967 | 70 | 0.3869 | 154 |
| **UPEC101 vs. *S. marcescens*** | < 0.0001 | 235 | < 0.0001 | 235 | < 0.0001 | 241 | < 0.0001 | 157 | < 0.0001 | 406 | < 0.0001 | 399 | < 0.0001 | 70 | < 0.0001 | 154 |
| **UPEC101 vs. *P. mirabilis*** | 0.0213 | 235 | > 0.9999 | 235 | 0.9998 | 241 | 0.9999 | 157 | > 0.9999 | 406 | > 0.9999 | 399 | 0.9061 | 70 | 0.9822 | 154 |
| **UPEC101 vs. *K. pneumoniae*** | 0.1189 | 235 | 0.9444 | 235 | 0.9998 | 241 | 0.9999 | 157 | > 0.9999 | 406 | > 0.9999 | 399 | > 0.9999 | 70 | 0.2495 | 154 |
| **UPEC101 vs. *Enterobacter spp.*** | < 0.0001 | 235 | > 0.9999 | 235 | 0.0361 | 241 | 0.3767 | 157 | 0.6023 | 406 | 0.0387 | 399 | 0.011 | 70 | 0.85 | 154 |
| **UPEC101 vs. *S. saprophyticus*** | < 0.0001 | 235 | < 0.0001 | 235 | 0.0007 | 241 | < 0.0001 | 157 | 0.4954 | 406 | < 0.0001 | 399 | 0.011 | 70 | < 0.0001 | 154 |
| **UPEC101 vs. *S. aureus*** | < 0.0001 | 235 | < 0.0001 | 235 | < 0.0001 | 241 | < 0.0001 | 157 | < 0.0001 | 406 | < 0.0001 | 399 | < 0.0001 | 70 | < 0.0001 | 154 |
| **UPEC101 vs. *P. rettgeri*** | 0.9587 | 235 | 0.9998 | 235 | > 0.9999 | 241 | 0.9999 | 157 | > 0.9999 | 406 | > 0.9999 | 399 | 0.9967 | 70 | 0.2495 | 154 |
| **UPEC101 vs. *M. morganii*** | > 0.9999 | 235 | 0.8416 | 235 | 0.9998 | 241 | 0.6789 | 157 | 0.9969 | 406 | 0.9803 | 399 | 0.9998 | 70 | 0.0817 | 154 |
| **UPEC102 vs. *S. marcescens*** | < 0.0001 | 235 | < 0.0001 | 235 | < 0.0001 | 241 | < 0.0001 | 157 | < 0.0001 | 406 | < 0.0001 | 399 | < 0.0001 | 70 | < 0.0001 | 154 |
| **UPEC102 vs*. P. mirabilis*** | 0.5491 | 235 | 0.9998 | 235 | 0.8966 | 241 | 0.9999 | 157 | 0.2587 | 406 | 0.983 | 399 | > 0.9999 | 70 | 0.9967 | 154 |
| **UPEC102 vs. *K. pneumoniae*** | 0.9021 | 235 | 0.5474 | 235 | 0.8966 | 241 | 0.9999 | 157 | 0.8732 | 406 | 0.9998 | 399 | 0.9758 | 70 | > 0.9999 | 154 |
| **UPEC102 vs. *Enterobacter spp.*** | 0.0054 | 235 | 0.9783 | 235 | 0.0015 | 241 | 0.0027 | 157 | 0.0002 | 406 | < 0.0001 | 399 | 0.2265 | 70 | 0.0017 | 154 |
| **UPEC102 vs. *S. saprophyticus*** | < 0.0001 | 235 | < 0.0001 | 235 | 0.0204 | 241 | 0.0012 | 157 | > 0.9999 | 406 | 0.0003 | 399 | 0.0002 | 70 | < 0.0001 | 154 |
| **UPEC102 vs. *S. aureus*** | < 0.0001 | 235 | < 0.0001 | 235 | < 0.0001 | 241 | < 0.0001 | 157 | < 0.0001 | 406 | < 0.0001 | 399 | < 0.0001 | 70 | < 0.0001 | 154 |
| **UPEC102 vs. *P. rettgeri*** | > 0.9999 | 235 | > 0.9999 | 235 | 0.9895 | 241 | 0.9999 | 157 | 0.2212 | 406 | 0.4785 | 399 | 0.5767 | 70 | > 0.9999 | 154 |
| **UPEC102 vs. *M. morganii*** | > 0.9999 | 235 | 0.9968 | 235 | > 0.9999 | 241 | > 0.9999 | 157 | 0.9761 | 406 | > 0.9999 | 399 | 0.7668 | 70 | > 0.9999 | 154 |
| ***S. marcescens* vs. *P. mirabilis*** | < 0.0001 | 235 | < 0.0001 | 235 | < 0.0001 | 241 | < 0.0001 | 157 | < 0.0001 | 406 | < 0.0001 | 399 | < 0.0001 | 70 | < 0.0001 | 154 |
| ***S. marcescens* vs. *K. pneumoniae*** | < 0.0001 | 235 | < 0.0001 | 235 | < 0.0001 | 241 | < 0.0001 | 157 | < 0.0001 | 406 | < 0.0001 | 399 | < 0.0001 | 70 | < 0.0001 | 154 |
| ***S. marcescens* vs. *Enterobacter spp.*** | < 0.0001 | 235 | < 0.0001 | 235 | < 0.0001 | 241 | < 0.0001 | 157 | 0.0004 | 406 | < 0.0001 | 399 | 0.0263 | 70 | < 0.0001 | 154 |
| ***S. marcescens* vs. *S. saprophyticus*** | < 0.0001 | 235 | < 0.0001 | 235 | < 0.0001 | 241 | < 0.0001 | 157 | < 0.0001 | 406 | < 0.0001 | 399 | < 0.0001 | 70 | < 0.0001 | 154 |
| ***S. marcescens* vs. *S. aureus*** | < 0.0001 | 235 | < 0.0001 | 235 | < 0.0001 | 241 | < 0.0001 | 157 | < 0.0001 | 406 | < 0.0001 | 399 | < 0.0001 | 70 | < 0.0001 | 154 |
| ***S. marcescens* vs. *P. rettgeri*** | < 0.0001 | 235 | < 0.0001 | 235 | < 0.0001 | 241 | < 0.0001 | 157 | < 0.0001 | 406 | < 0.0001 | 399 | < 0.0001 | 70 | < 0.0001 | 154 |
| ***S. marcescens* vs. *M. morganii*** | < 0.0001 | 235 | < 0.0001 | 235 | < 0.0001 | 241 | < 0.0001 | 157 | < 0.0001 | 406 | < 0.0001 | 399 | < 0.0001 | 70 | < 0.0001 | 154 |
| ***P. mirabilis* vs. *K. pneumoniae*** | > 0.9999 | 235 | 0.9783 | 235 | > 0.9999 | 241 | > 0.9999 | 157 | 0.9996 | 406 | > 0.9999 | 399 | 0.7668 | 70 | 0.9822 | 154 |
| ***P. mirabilis* vs. *Enterobacter spp*.** | 0.9021 | 235 | > 0.9999 | 235 | 0.3329 | 241 | 0.0497 | 157 | 0.7057 | 406 | 0.0239 | 399 | 0.5767 | 70 | 0.0817 | 154 |
| ***P. mirabilis* vs. *S. saprophyticus*** | < 0.0001 | 235 | < 0.0001 | 235 | < 0.0001 | 241 | < 0.0001 | 157 | 0.3926 | 406 | < 0.0001 | 399 | < 0.0001 | 70 | < 0.0001 | 154 |
| ***P. mirabilis* vs*. S. aureus*** | < 0.0001 | 235 | < 0.0001 | 235 | < 0.0001 | 241 | < 0.0001 | 157 | < 0.0001 | 406 | < 0.0001 | 399 | < 0.0001 | 70 | < 0.0001 | 154 |
| ***P. mirabilis* vs*. P. rettgeri*** | 0.6884 | 235 | 0.9986 | 235 | > 0.9999 | 241 | > 0.9999 | 157 | > 0.9999 | 406 | 0.9993 | 399 | 0.2265 | 70 | 0.9822 | 154 |
| ***P. mirabilis* vs. *M. morganii*** | 0.1905 | 235 | 0.737 | 235 | 0.8966 | 241 | 0.9914 | 157 | 0.9905 | 406 | 0.9946 | 399 | 0.3832 | 70 | 0.85 | 154 |
| ***K. pneumoniae* vs. *Enterobacter spp.*** | 0.5491 | 235 | 0.9998 | 235 | 0.3329 | 241 | 0.0497 | 157 | 0.1315 | 406 | 0.0056 | 399 | 0.0044 | 70 | 0.0007 | 154 |
| ***K. pneumoniae* vs. *S. saprophyticus*** | < 0.0001 | 235 | < 0.0001 | 235 | < 0.0001 | 241 | < 0.0001 | 157 | 0.9485 | 406 | < 0.0001 | 399 | 0.0263 | 70 | < 0.0001 | 154 |
| ***K. pneumoniae* vs. *S. aureus*** | < 0.0001 | 235 | < 0.0001 | 235 | < 0.0001 | 241 | < 0.0001 | 157 | < 0.0001 | 406 | < 0.0001 | 399 | < 0.0001 | 70 | < 0.0001 | 154 |
| ***K. pneumoniae* vs. *P. rettgeri*** | 0.9587 | 235 | 0.4192 | 235 | > 0.9999 | 241 | > 0.9999 | 157 | 0.9992 | 406 | 0.9756 | 399 | 0.9998 | 70 | > 0.9999 | 154 |
| ***K. pneumoniae* vs. *M. morganii*** | 0.5491 | 235 | 0.0392 | 235 | 0.8966 | 241 | 0.9914 | 157 | > 0.9999 | 406 | > 0.9999 | 399 | > 0.9999 | 70 | > 0.9999 | 154 |
| ***Enterobacter spp*. vs*. S. saprophyticus*** | < 0.0001 | 235 | < 0.0001 | 235 | < 0.0001 | 241 | < 0.0001 | 157 | 0.0004 | 406 | < 0.0001 | 399 | < 0.0001 | 70 | < 0.0001 | 154 |
| ***Enterobacter spp*. vs. *S. aureus*** | < 0.0001 | 235 | < 0.0001 | 235 | < 0.0001 | 241 | < 0.0001 | 157 | < 0.0001 | 406 | < 0.0001 | 399 | < 0.0001 | 70 | < 0.0001 | 154 |
| ***Enterobacter spp*. vs. *P. rettgeri*** | 0.011 | 235 | 0.9444 | 235 | 0.1259 | 241 | 0.0497 | 157 | 0.7537 | 406 | 0.3245 | 399 | 0.0002 | 70 | 0.0007 | 154 |
| ***Enterobacter spp*. vs. *M. morganii*** | 0.0005 | 235 | 0.3594 | 235 | 0.0015 | 241 | 0.0005 | 157 | 0.0473 | 406 | 0.0002 | 399 | 0.0006 | 70 | < 0.0001 | 154 |
| ***S. saprophyticus* vs. *S. aureus*** | < 0.0001 | 235 | < 0.0001 | 235 | < 0.0001 | 241 | < 0.0001 | 157 | < 0.0001 | 406 | < 0.0001 | 399 | < 0.0001 | 70 | < 0.0001 | 154 |
| ***S. saprophyticus* vs. *P. rettgeri*** | < 0.0001 | 235 | < 0.0001 | 235 | 0.0001 | 241 | < 0.0001 | 157 | 0.3447 | 406 | < 0.0001 | 399 | 0.2265 | 70 | < 0.0001 | 154 |
| ***S. saprophyticus* vs. *M. morganii*** | < 0.0001 | 235 | < 0.0001 | 235 | 0.0204 | 241 | 0.006 | 157 | 0.9945 | 406 | 0.0002 | 399 | 0.1206 | 70 | < 0.0001 | 154 |
| ***S. aureus* vs. *P. rettgeri*** | < 0.0001 | 235 | < 0.0001 | 235 | < 0.0001 | 241 | < 0.0001 | 157 | < 0.0001 | 406 | < 0.0001 | 399 | < 0.0001 | 70 | < 0.0001 | 154 |
| ***S. aureus* vs. *M. morganii*** | < 0.0001 | 235 | < 0.0001 | 235 | < 0.0001 | 241 | < 0.0001 | 157 | < 0.0001 | 406 | < 0.0001 | 399 | < 0.0001 | 70 | < 0.0001 | 154 |
| ***P. rettgeri* vs. *M. morganii*** | > 0.9999 | 235 | 0.9994 | 235 | 0.9895 | 241 | 0.9914 | 157 | 0.9846 | 406 | 0.6076 | 399 | > 0.9999 | 70 | > 0.9999 | 154 |
| **F value** | 234.5 | | 272.9 | | 144.8 | | 165.9 | | 48.22 | | 97.93 | | 57.72 | | 206.9 | |

**Supplementary Table 5: Comparison of the antimicrobial efficacy of fresh (fAM) and cryopreserved AM (cAM) homogenates.** fAM homogenate elicits the greatest antimicrobial effect, while the cryopreservation decreases the antimicrobial efficacy of cAM (1 week at –80°C), cAM (10 weeks at –80°C) and cAM (10 weeks at –20°C) homogenates. Larger volumes of homogenates (10 µl) have greater antimicrobial effect than smaller volumes (5 µl).

|  | **AM homogenate** | | | | | | | |
| --- | --- | --- | --- | --- | --- | --- | --- | --- |
|  |  |  |  |  |  |  |  |  |
|  | **fAM** | | **cAM**  **(1 week at** –**80°C)** | | **cAM**  **(10 weeks at** –**80°C)** | | **cAM**  **(10 weeks at** –**20°C)** | |
| **Volume of AM homogenate used** | **5 µl** | **10 µl** | **5 µl** | **10 µl** | **5 µl** | **10 µl** | **5 µl** | **10 µl** |
| **Gram-positive tested strains** | | | | | | | | |
| ***S. aureus***  Mean diameter of the antimicrobial zone ± standard error (mm) | 16.0 ± 0.2 | 19.0 ± 0.3 | 12.3 ± 0.4 | 16.1 ± 0.4 | 10.2 ± 0.6 | 14.5 ± 0.3 | 14.3 ± 0.5 | 17.7 ± 0.4 |
| *Decrease in the range of the antimicrobial zone in comparison to the mean diameter of the antimicrobial zone due to the application of fAM homogenate (%)* |  |  | 23.1% | 14.8% | 36.3% | 23.3% | 10.6% | 6.3% |
| ***S. saprophyticus***  Mean diameter of the antimicrobial zone ± standard error (mm) | 8.2 ± 0.4 | 10.2 ± 0.4 | 6.1 ± 0.3 | 8.5 ± 0.6 | 4.3 ± 0.5 | 8.1 ± 0.5 | 6.8 ± 0.7 | 9.3 ± 0.3 |
| *Decrease in the range of the antimicrobial zone in comparison to the mean diameter of the antimicrobial zone due to the application of fAM homogenate (%)* |  |  | 25.6% | 16.7% | 47.6% | 20.6% | 17.1% | 8.8% |
| **Gram-negative tested strains** | | | | | | | | |
| **UPEC DL94**  Mean diameter of the antimicrobial zone ± standard error (mm) | 7.6 ± 0.2 | 9.5 ± 0.2 | 5.3 ± 0.2 | 7.7 ± 0.3 | 5.5 ± 0.2 | 7.0 ± 0.2 | 5.7 ± 0.4 | 8.5 ± 0.2 |
| *Decrease in the range of the antimicrobial zone in comparison to the mean diameter of the antimicrobial zone due to the application of fAM homogenate (%)* |  |  | 30.3% | 18.9% | 27.6% | 26.3% | 25.9% | 10.5% |
| ***E. coli* DH5α**  Mean diameter of the antimicrobial zone ± standard error (mm) | 6.1 ± 0.2 | 8.2 ± 0.2 | 5.3 ± 0.2 | 7.2 ± 0.2 | 3.6 ± 0.4 | 5.4 ± 0.4 | 4.8 ± 0.2 | 7.1 ± 0.1 |
| *Decrease in the range of the antimicrobial zone in comparison to the mean diameter of the antimicrobial zone due to the application of fAM homogenate (%)* |  |  | 13.1% | 12.2% | 41.0% | 34.1% | 21.3% | 13.4% |
| ***M. morganii***  Mean diameter of the antimicrobial zone ± standard error (mm) | 6.3 ± 0.1 | 8.1 ± 0.2 | 4.9 ± 0.1 | 6.9 ± 0.2 | 3.7 ± 0.3 | 5.9 ± 0.2 | 5.0 ± 0.5 | 7.2 ± 0.1 |
| *Decrease in the range of the antimicrobial zone in comparison to the mean diameter of the antimicrobial zone due to the application of fAM homogenate (%)* |  |  | 22.2% | 14.8% | 41.3% | 27.2% | 20.6% | 11.1% |
| ***P. rettgeri***  Mean diameter of the antimicrobial zone ± standard error (mm) | 6.1 ± 0.2 | 7.8 ± 0.2 | 4.5 ± 0.3 | 6.4 ± 0.1 | 3.1 ± 0.3 | 4.9 ± 0.4 | 5.2 ± 0.4 | 7.0 ± 0.1 |
| *Decrease in the range of the antimicrobial zone in comparison to the mean diameter of the antimicrobial zone due to the application of fAM homogenate (%)* |  |  | 26.2% | 17.9% | 49.2% | 37.2% | 14.8% | 10.3% |
| **UPEC DL102**  Mean diameter of the antimicrobial zone ± standard error (mm) | 6.1 ± 0.1 | 7.8 ± 0.2 | 4.9 ± 0.2 | 6.8 ± 0.2 | 4.4 ± 0.1 | 6.0 ± 0.2 | 3.8 ± 0.3 | 6.9 ± 0.1 |
| *Decrease in the range of the antimicrobial zone in comparison to the mean diameter of the antimicrobial zone due to the application of fAM homogenate (%)* |  |  | 19.7% | 12.8% | 27.9% | 23.1% | 45.9% | 11.5% |
| **UPEC DL90**  Mean diameter of the antimicrobial zone ± standard error (mm) | 5.8 ± 0.2 | 7.6 ± 0.1 | 3.4 ± 0.2 | 5.3 ± 0.3 | 1.8 ± 0.3 | 4.1 ± 0.3 | 4.3 ± 0.3 | 6.0 ± 0.2 |
| *Decrease in the range of the antimicrobial zone in comparison to the mean diameter of the antimicrobial zone due to the application of fAM homogenate (%)* |  |  | 41.4% | 30.3% | 69.0% | 46.1% | 25.9% | 21.1% |
| **UPEC DL101**  Mean diameter of the antimicrobial zone ± standard error (mm) | 6.5 ± 0.3 | 7.6 ± 0.2 | 4.6 ± 0.1 | 6.1 ± 0.2 | 3.2 ± 0.3 | 5.3 ± 0.3 | 4.5 ± 0.2 | 6.0 ± 0.2 |
| *Decrease in the range of the antimicrobial zone in comparison to the mean diameter of the antimicrobial zone due to the application of fAM homogenate (%)* |  |  | 29.2% | 19.7% | 50.8% | 30.3% | 30.8% | 21.1% |
| ***P. mirabilis***  Mean diameter of the antimicrobial zone ± standard error (mm) | 5.4 ± 0.2 | 7.5 ± 0.2 | 4.3 ± 0.2 | 6.4 ± 0.1 | 3.1 ± 0.3 | 5.4 ± 0.2 | 3.5 ± 0.5 | 6.5 ± 0.1 |
| *Decrease in the range of the antimicrobial zone in comparison to the mean diameter of the antimicrobial zone due to the application of fAM homogenate (%)* |  |  | 20.4% | 14.7% | 42.6% | 28.0% | 35.2% | 13.3% |
| **UPEC DL88**  Mean diameter of the antimicrobial zone ± standard error (mm) | 6.0 ± 0.2 | 7.3 ± 0.2 | 4.6 ± 0.1 | 6.5 ± 0.1 | 3.7 ± 0.3 | 5.3 ± 0.4 | 4.5 ± 0.4 | 6.7 ± 0.2 |
| *Decrease in the range of the antimicrobial zone in comparison to the mean diameter of the antimicrobial zone due to the application of fAM homogenate (%)* |  |  | 23,3% | 11,0% | 38,3% | 27,4% | 25,0% | 8,2% |
| ***Enterobacter spp.***  Mean diameter of the antimicrobial zone ± standard error (mm) | 4.9 ± 0.2 | 7.3 ± 0.2 | 3.5 ± 0.2 | 5.1 ± 0.3 | 2.1 ± 0.2 | 3.8 ± 0.3 | 2.2 ± 0.4 | 5.3 ± 0.2 |
| *Decrease in the range of the antimicrobial zone in comparison to the mean diameter of the antimicrobial zone due to the application of fAM homogenate (%)* |  |  | 28.6% | 30.1% | 57.1% | 47.9% | 55.1% | 27.4% |
| ***K. pneumoniae***  Mean diameter of the antimicrobial zone ± standard error (mm) | 5.6 ± 0.1 | 7.1 ± 0.2 | 4.3 ± 0.1 | 6.4 ± 0.2 | 3.5 ± 0.3 | 5.6 ± 0.3 | 4.7 ± 0.2 | 7.0 ± 0.1 |
| *Decrease in the range of the antimicrobial zone in comparison to the mean diameter of the antimicrobial zone due to the application of fAM homogenate (%)* |  |  | 23.3% | 9.9% | 37.5% | 21.1% | 16.1% | 1.4% |
| ***S. marcescens***  Mean diameter of the antimicrobial zone ± standard error (mm) | – | – | – | – | – | – | – | – |
| **Average antimicrobial zone for all susceptible strains**  Mean diameter of the antimicrobial zone ± standard error (mm) | **7.0 ± 0.2** | **8.8 ± 0.2** | **5.2 ± 0.2** | **7.3 ± 0.2** | **4.0 ± 0.3** | **6.3 ± 0.3** | **5.3 ± 0.4** | **7.8 ± 0.2** |
| *Decrease in the range of the antimicrobial zone in comparison to the mean diameter of the antimicrobial zone due to the application of fAM homogenate (%)* |  |  | 25.1 ± 2.0 % | 17.2 ± 2.0 % | 43.5 ± 3.5 % | 30.2 ± 2.8 % | 26.4 ± 3.9 % | 12.7 ± 2.1 % |


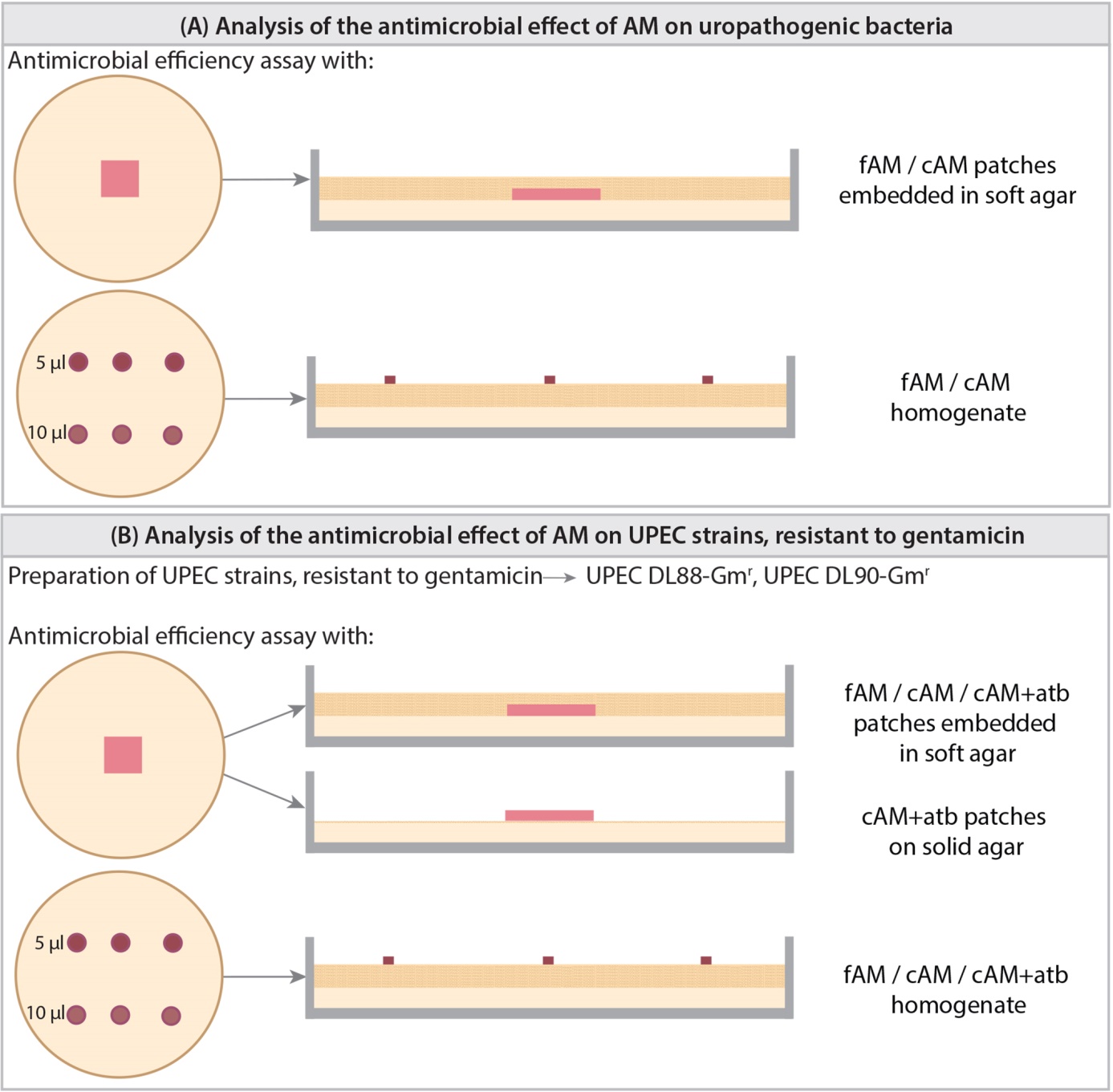


**Supplementary Figure 1:** **Experimental design of this study.** The newly developed antimicrobial efficacy assays to test the effect of fAM, cAM or cAM+atb patches and fAM, cAM or cAM+atb homogenates on A) uropathogenic bacteria and B) UPEC strains, resistant to gentamicin.
